# SF3B1 homeostasis is critical for survival and therapeutic response in T cell leukemia

**DOI:** 10.1126/sciadv.abj8357

**Published:** 2022-01-21

**Authors:** Cuijuan Han, Alireza Khodadadi-Jamayran, Adam H. Lorch, Qi Jin, Valentina Serafin, Ping Zhu, Yuliya Politanska, Limin Sun, Blanca T. Gutierrez-Diaz, Marina V. Pryzhkova, Hiam Abdala-Valencia, Elizabeth Thomas Bartom, Barbara Buldini, Giuseppe Basso, Sadanandan E. Velu, Kavitha Sarma, Basil B. Mattamana, Byoung-Kyu Cho, Rebecca C. Obeng, Young Ah Goo, Philip W. Jordan, Aristotelis Tsirigos, Yalu Zhou, Panagiotis Ntziachristos

**Affiliations:** 1 Department of Biochemistry and Molecular Genetics, Northwestern University, Chicago, IL, USA.; 2 Simpson Querrey Institute for Epigenetics, Northwestern University Feinberg School of Medicine, Chicago, IL, USA.; 3 Applied Bioinformatics Laboratories, Office of Science and Research, New York University School of Medicine, New York, NY, USA.; 4 Division of Pediatric Hematology, Oncology and Stem Cell Transplant, Maternal and Child Health Department, Padua University, Padova, Italy.; 5 H3 Biomedicine Inc., Cambridge, MA, USA.; 6 Department of Medicine, Northwestern University Feinberg School of Medicine, Chicago, IL, USA.; 7 Department of Biochemistry and Molecular Biology, Bloomberg School of Public Health, Johns Hopkins University, Baltimore, MD, USA.; 8 Department of Chemistry, University of Alabama at Birmingham, Birmingham, AL, USA.; 9 Gene Expression and Regulation Program, The Wistar Institute, Philadelphia, PA, USA.; 10 Epigenetics Institute, University of Pennsylvania, Philadelphia, PA, USA.; 11 Proteomics Center of Excellence, Northwestern University, Evanston, IL, USA.; 12 Department of Pathology, Northwestern University Feinberg School of Medicine, Chicago, IL, USA.; 13 Department of Pathology and Laura & Isaac Perlmutter Cancer Center, NYU School of Medicine, New York, NY, USA.; 14 Institute for Computational Medicine, NYU School of Medicine, New York, NY, USA.; 15 Robert H. Lurie Comprehensive Cancer Center, Northwestern University, Chicago, IL, USA.

## Abstract

The production of noncanonical mRNA transcripts is associated with cell transformation. Driven by our previous findings on the sensitivity of T cell acute lymphoblastic leukemia (T-ALL) cells to SF3B1 inhibitors, we identified that SF3B1 inhibition blocks T-ALL growth in vivo with no notable associated toxicity. We also revealed protein stabilization of the U2 complex component SF3B1 via deubiquitination. Our studies showed that SF3B1 inhibition perturbs exon skipping, leading to nonsense-mediated decay and diminished levels of DNA damage response–related transcripts, such as the serine/threonine kinase *CHEK2*, and impaired DNA damage response. We also identified that SF3B1 inhibition leads to a general decrease in R-loop formation. We further demonstrate that clinically used SF3B1 inhibitors synergize with CHEK2 inhibitors and chemotherapeutic drugs to block leukemia growth. Our study provides the proof of principle for posttranslational regulation of splicing components and associated roles and therapeutic implications for the U2 complex in T cell leukemia.

## Teaser

The splicing factor SF3B1 is regulated at the protein level and is a therapeutic target in T cell leukemia.

## INTRODUCTION

Alternative mRNA splicing affects more than 95% of multiexon pre-mRNAs in higher eukaryotes (*1*). Aberrant splicing is a hallmark of cancer and a potential target for cancer therapeutics and is dictated by aberrant expression level and mutations of splicing factors that can be found in at least 50% of patient cases (*2*). SF3B1, a key U2 spliceosome component, regulates the early splicing stages by recognizing the branch point intronic sequence, which is affected by hotspot mutations common in patients with chronic lymphocytic leukemia (CLL) and myelodysplastic syndromes (MDSs) and has been associated with aberrant splicing in hematological malignancies (*3*–*7*). Mutant SF3B1 promotes the usage of aberrant branch point sequences, resulting in the global selection of cryptic 3′ splice sites and tumorigenesis mainly through MYC stabilization (*8*, *9*). Recently, reports indicated that mutations in SF3B1 lead to the accumulation of R-loops, a chromosome structure that contains one strand of DNA and a DNA/RNA hybrid and can lead to increased DNA damage, if left unrepaired (*10*, *11*). In addition, an increasing number of recent studies have shown the emerging role of expression alterations of splicing factors in cancer (*12*, *13*). Numerous studies have shown that active transcription and splicing are functionally coupled (*14*–*16*). Splicing factors of the serine/arginine-rich family, such as SRSF2, have been shown to interact with components of the transcriptional machinery to mediate transcriptional activation (*14*, *17*). Conversely, the rate of elongation, the type of promoter, and transcription-related factors have been shown to affect alternative splicing (*18*, *19*). Tumors with high levels of the transcription factor and transcriptional amplifier MYC are highly dependent on splicing regulation, which translates to increased sensitivity to splicing perturbations (*20*).

T cell acute lymphoblastic leukemia (T-ALL) is an aggressive disease that accounts for approximately 15% of pediatric and 25% of adult ALL cases (*21*–*26*). Standard- and high-dose chemotherapy is the modern first-line treatment for T-ALL, and treatment regimens can last up to 3 years. We have reported that T-ALL presents with an increased number of epigenetic, transcriptional, and splicing alterations and is thus a suitable model for the study of splicing and transcription (*26*, *27*). We demonstrated aberrant exon skipping that affects critical pathways such as the proteasome pathway despite the near absence of splicing factor mutations in T-ALL (*24*, *26*, *28*). Past studies have also shown that inhibition of SF3B1 previously evaluated in the clinic against MDS, acute myeloid leukemia (AML), and chronic monomyelocytic leukemia (CMML) (*29*–*31*) blocks the growth of leukemia cells and exhibits relatively low toxicity toward normal CD34^+^ hematopoietic progenitors (*26*).

We identified that the SF3B1 protein is highly expressed in T-ALL via active deubiquitination by ubiquitin-specific peptidase 7 (USP7), and that inhibition of USP7 activity can lead to SF3B1 degradation. We also showed that SF3B1 inhibition blocks active transcription, leading to changes in R-loops, and causes exon-skipping changes affecting DNA repair transcripts, such as *CHEK2*, leading to nonsense-mediated decay (NMD) of the transcript. SF3B1 inhibition enhances sensitivity to chemotherapy and CHEK2 inhibitors. Our findings suggest that the leukemia cells that depend on high SF3B1 levels might stem from the role of SF3B1 in controlling oncogenic transcription in addition to splicing, leading to its impact on drug resistance in high-risk (HR) cancers.

## RESULTS

### SF3B1 inhibition blocks leukemia growth

**Fig. 1.**
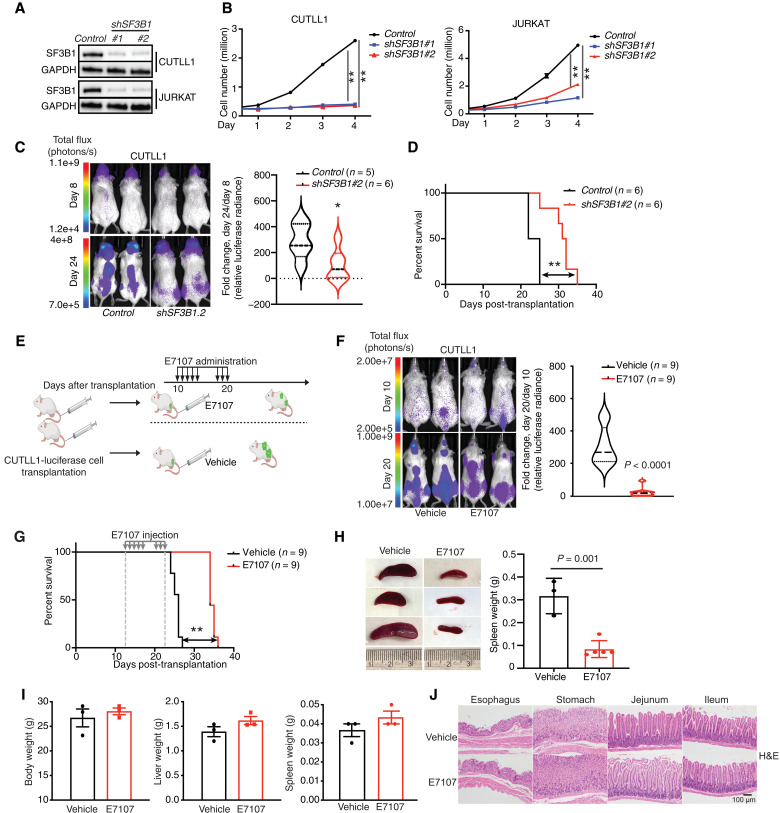
SF3B1 silencing or inhibition blocks growth of T cell leukemia. (**A**) Immunoblot indicating SF3B1 depletion in CUTLL1 and JURKAT cells. GAPDH, glyceraldehyde-3-phosphate dehydrogenase. (**B**) CUTLL1 and JURKAT cells were counted at the indicated time points, and cell growth was plotted as shown for the *shSF3B1#1*- and *shSF3B1#2*-expressing cell populations (*n* = 3). (**C**) Luciferase-expressing CUTLL1 cells were transduced with *control* or *shSF3B1#2* and injected intravenously (retro-orbitally) into immunocompromised mice. Luminescence analysis for representative mice on days 8 and 24 (left) and luminescence intensity fold change between days 8 and 24 are shown (right: *control*, *n* = 5; *shSF3B1#2*, *n* = 6). (**D**) Survival curve analysis of mice from (C). *P ≤ 0.05 and **P ≤ 0.01. (**E**) E7107 treatment schema in the xenograft model. (**F**) Luciferase-expressing CUTLL1 cells were intravenously (tail vein) injected into immunocompromised mice followed by 8 days of E7107 administration (at 5 mg/kg per day) starting on the 10th day after transplantation. Luminescence images for representative mice on days 10 and 20 (left) and luminescence intensity fold change between days 10 and 20 are shown (right: vehicle, *n* = 9; E7107, *n* = 9). (**G**) Survival analysis of mice from (F). (**H**) Mouse spleen size (left) and weight (right) upon E7107 treatment of the NOTCH1-δE-Cherry^+^ retroviral T-ALL model [vehicle (*n* = 3), E7107 (*n* = 5)]. (**I**) Representative mice body weight, liver weight, and spleen weight analysis upon treatment with E7107. (**J**) Representative hematoxylin and eosin (H&E) staining of esophagus, stomach, jejunum, and ileum (×200 magnification).

The U2 small nuclear ribonucleoprotein (snRNP) complex is the main chemically targetable splicing complex and consists of splicing factor 3A/B family members (SF3A1, SF3A2, SF3A3, SF3B1, SF3B2, SF3B3, SF3B4, SF3B5, SF3B6, SF3B7, and SF3B14), plant-homeodomain finger-like domain factor 5A (PHF5A), and SF1 (*32*). We have previously demonstrated the sensitivity of T-ALL cells to splicing perturbations, including SRSF6 silencing and SF3B1 inhibition (*26*). We have shown that SRSF6 silencing leads to perturbation of exon skipping affecting proteasomal, cell cycle, and RNA biology–related transcripts. To address the functional role of SF3B1 and associated mechanisms behind T-ALL sensitivity to splicing perturbations, we silenced *SF3B1* in T-ALL cell lines using short hairpin RNAs (shRNAs). The cell proliferation rate was significantly decreased upon *SF3B1* silencing (Fig. 1, A and B), accompanied by an increase in apoptotic cell death and G_2_-M cell cycle arrest (fig. S1, A and B). T-ALL cell lines present with sensitivity to E7107 inhibitor at an IC_50_ (median inhibitory concentration) concentration at the nanomolar range (fig. S1C), similarly to T-ALL diagnostic patient samples (fig. S1D). Treatment with the SF3B1 inhibitor E7107 also showed a G_2_-M cell cycle arrest and a slight increase in apoptosis (fig. S1, E to H). To further assess the role of SF3B1 in disease progression in vivo, we silenced *SF3B1* in luciferase-expressing CUTLL1 cells and transplanted the cells into immunocompromised mice. *SF3B1* silencing led to a significantly reduced tumor burden and prolonged mouse survival (Fig. 1, C and D). We further examined whether chemical inhibition of SF3B1 activity could suppress tumor growth in vivo. E7107 treatment resulted in a decreased disease burden and a survival benefit compared to vehicle (Fig. 1, E to G). The use of an independent mouse model of T-ALL using overexpression of an oncogenic truncated allele of NOTCH1 transcription factor (NOTCH1-ΔE) in hematopoietic progenitors (*33*), coupled to transplantation into immunocompromised mouse recipients, also showed inhibition of tumor growth upon E7107 treatment, as indicated by the reduction in spleen size (Fig. 1H). Our previous studies showed that the effect of SF3B1 inhibition presents with reduced toxicity in hematopoietic progenitor cells, such as CD34^+^ cells (*26*), and our present in vivo toxicity analysis failed to detect significant toxicity associated with body or organ weight, blood populations, goblet cell metaplasia, or other gastrointestinal toxicity such as the one previously demonstrated for gamma secretase inhibitor drugs used to block NOTCH1 activity in T-ALL therapy (Fig. 1, I and J, fig. S1I, and table S1) (*34*). Together, our data demonstrate that SF3B1 levels and activity are critical for leukemia cell survival.

### SF3B1 is posttranslationally regulated in T cell leukemia

**Fig. 2.**
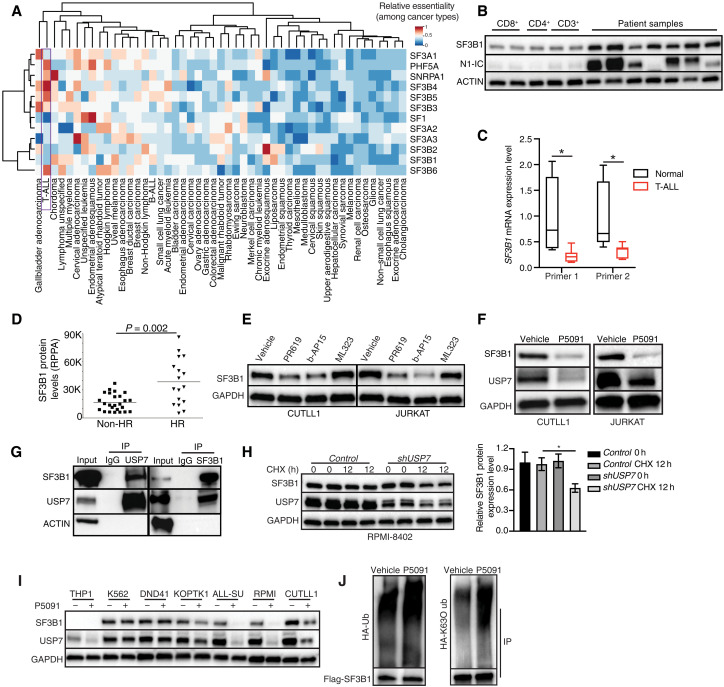
SF3B1 is posttranslationally regulated in T cell leukemia. (**A**) Relative essentiality of the U2 splicing complex components across different types of cancers. Essentiality data were obtained from the Project Achilles CRISPR-Cas9 screening dataset for 563 cancer cell lines. (**B**) Immunoblot showing SF3B1 protein level in patients versus T cells [see fig. S2D for the quantification of protein levels, (N1-IC, NOTCH1 intracellular domain)]. (**C**) Reverse transcription reaction coupled to reverse transcription polymerase chain reaction (RT-PCR) for the SF3B1 mRNA expression in T-ALL versus T cells in human sample (*n* = 3). *P ≤ 0.05. (**D**) RPPA analysis for SF3B1 protein levels in HR (*n* = 17) versus non-HR (*n* = 25) patient cases. (**E**) Immunoblot indicating SF3B1 protein expression level in CUTLL1 and JURKAT cells treated with 10 μM PR619, 2 μM b-AP15, or 200 nM ML323 (24 hours). GAPDH is used as the loading control. (**F**) SF3B1 protein level upon treatment of CUTLL1 and JURKAT with 10 μM P5091 for 24 hours. (**G**) Representative coimmunoprecipitation analysis of USP7 (left) and SF3B1 (right) to evaluate the interaction between USP7 and SF3B1 in CUTLL1 cells. IP, immunoprecipitation; IgG, immunoglobulin G. (**H**) Representative immunoblot of SF3B1 protein expression upon treatment with cycloheximide (CHX) (10 μg/ml) in *control*- and *shUSP7*-expressing RPMI-8402 cells for 12 hours. Quantification of protein levels is shown (right). (**I**) Representative immunoblot of SF3B1 protein expression upon treatment of cells with 10 μM P5091 (24 hours). (**J**) Immunoprecipitation of Flag-tagged SF3B1 coupled to immunoblot analysis for detection of SF3B1 ubiquitination in 293T cells cotransfected with hemagglutinin (HA)–tagged or HA-K63O (only K63 can be ubiquitinated) ubiquitin (Ub) construct and Flag-SF3B1.

Driven by the sensitivity of T-ALL cells to SF3B1 inhibitors despite the general absence of SF3B1 mutations (*26*), we sought out to further investigate alterations in the levels and genetic status of SF3B1 in T-ALL. Our past study and data from the pediatric cancer genome project show that there are very few spliceosome mutations in T-ALL, in contrast to other hematological and solid tumors (*26*). To assess the essentiality of U2 family members in cancer, we analyzed genetic functional screens and gene expression data for 563 cell lines from solid and hematological tumors from the Cancer Dependency Map project (DepMap; https://depmap.org/portal/) (*35*), which contains about 560 cell lines including three T-ALL cell lines (SUPT1, PF382, and HSB2). The U2 family members were collectively critical for survival in T-ALL compared to other cancers (Fig. 2A) (*26*). Analysis of U2 family mRNA expression levels in the Cancer Cell Line Encyclopedia (CCLE) depository showed that SF3B1 was one of the most highly expressed U2 family members in T-ALL (fig. S2, A and B). We then examined the SF3B1, SF3A3, and PHF5A levels in a panel of physiological T cell subsets, including CD3^+^, CD4^+^, and CD8^+^ T cells, and seven T-ALL patients. SF3B1 protein—but not transcript—levels were significantly higher in human patients and a mouse model of T-ALL compared to healthy T lymphocytes (Fig. 2, B and C, and fig. S2, C to E). We then compared SF3B1 protein levels in patients of the most aggressive T-ALL background (HR (high-risk) disease), who either relapsed or did not respond to chemotherapy to non-HR patient cases. The SF3B1 protein levels, but not mRNA levels, were higher in HR patients compared to non-HR patients, suggesting that the SF3B1 protein levels might contribute to resistance to therapy (Fig. 2D and fig. S2F).

We and others have recently demonstrated the modification of spliceosome factors, such as hnRNPA1 and SRSF6, from ubiquitin-like proteins (UBLs) (*26*, *36*–*38*). Posttranslational regulation of splicing factors via deubiquitination by USP7 contributes to their high protein levels in leukemia (*26*). To investigate the potential role of USP7 in the control of SF3B1, we treated T-ALL cells with the global deubiquitinase inhibitor PR619 and the USP7 inhibitor P5091 as well as with compounds inhibiting the prooncogenic deubiquitinases USP1 (ML323) and USP14/UCHL5 (b-AP15) (*39*–*43*). The PR619 inhibitor reduced SF3B1 protein—and not mRNA—levels similarly to P5091 and b-AP15 (Fig. 2, E and F, and fig. S2, G and H). In contrast, treatment with the JQ1 inhibitor that affects MYC, a known transcriptional regulator of splicing genes, did not lead to changes in SF3B1 protein levels (fig. S2I), further suggesting that SF3B1 might be mainly regulated at the posttranslational level in T-ALL. Our evaluation of the interaction between SF3B1 and these deubiquitinases showed that USP7, but not USP14 or UCHL5, interacts with SF3B1 (Fig. 2G and fig. S2, J and K). We then further investigated the relationship between USP7 and SF3B1. Our studies showed that USP7 knockdown decreases SF3B1 protein level upon cycloheximide (CHX) treatment, but not the control group in RPMI-8402 and JURKAT cells (Fig. 2H and fig. S2L). SF3B1 protein showed shorter half-life upon CHX plus P5091 treatment compared to CHX single treatment (fig. S2, M and N). USP7 inhibition particularly affected nuclear SF3B1 levels compared to cytoplasmic SF3B1 levels, suggesting a critical role for USP7 in the functions of nuclear SF3B1 (fig. S2O). Comparison of SF3B1 and USP7 protein levels in T-ALL and other hematological (B-ALL and myeloid subtypes) and solid tumors (lung, breast, melanoma, and pancreatic adenocarcinomas) showed relatively high levels of SF3B1 and USP7 in leukemia cells compared to solid cancer cells (fig. S2P). This increased expression levels in leukemia associate with increased sensitivity of SF3B1 levels to USP7 inhibition in leukemia samples (Fig. 2I and fig. S2Q). We then analyzed SF3B1 ubiquitination upon treatment with P5091 to identify a notable increase in SF3B1 polyubiquitination (Fig. 2J, left). The use of ubiquitin mutants, which block the formation of polyubiquitin chains, showed the formation of K63-linked, but not K48-linked, polyubiquitin chains on SF3B1 (Fig. 2J, right, and fig. S2R). Together, our findings suggest that SF3B1 is regulated at the posttranslational level.

### DNA damage response transcripts are sensitive to SF3B1 perturbations

**Fig. 3.**
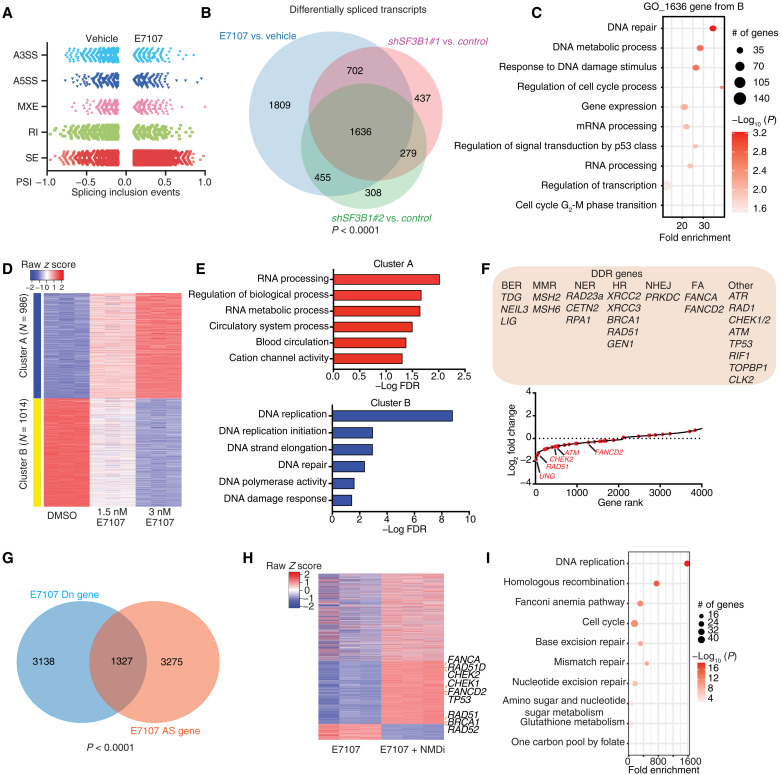
DNA damage response transcripts are sensitive to perturbation of SF3B1 levels. (**A**) Representation of E7107-induced splicing changes in CUTLL1 cells over 24-hour treatment. Events with false discovery rate (FDR) < 0.05 and percent spliced in (PSI) < 0.1 (10% of the transcripts of a given gene are affected) are presented. The alternative splicing events are classified into five categories: skipped exon (SE), retained intron (RI), mutually exclusive exon (MXE), alternative 5′ splice site (A5SS), and alternative 3′ splice site (A3SS). (**B**) Overlapping alternatively spliced transcripts (*n* = 1636) upon SF3B1 silencing (*shSF3B1#1/2*) and inhibition (3 nM E7107, 24 hours) (*P* < 0.001). (**C**) Gene ontology analysis for the transcript set from (B). (**D**) κ-Means clustering analysis of gene expression changes upon treatment of CUTLL1 cells with 1.5 and 3 nM E7107. (**E**) Gene ontology analysis of the two gene clusters from (D). (**F**) Main DNA damage response (DDR) genes with gene expression alterations upon E7107 treatment (1.5 and 3 nM, 24 hours, CUTLL1 cells) are presented (top). Waterfall plot of fold change in gene expression was induced by E7107 treatment (1.5 nM, 24 hours, CUTLL1 cells). Red dots represent DDR transcripts. (**G**) Overlap of alternative splicing (AS) genes in 3 nM E7107 (24 hours) versus vehicle and Dn-regulated genes in 3 nM E7107 (24 hours) versus vehicle. (**H**) Heatmap of changes in gene expression upon combinatorial treatment with E7107 plus NMD inhibitor (NMDi) (transcripts that are down-regulated upon E7107 treatment are shown, adjusted *P* < 0.01). (**I**) Gene ontology analysis of up-regulated genes in combination treatment of E7107 + NMDi versus E7107 only.

To gain an understanding of the role of SF3B1 in tumor growth, we aimed to characterize SF3B1-regulated splicing events. We performed transcriptomic analysis via paired-end RNA sequencing using the clinically used SF3B1 inhibitor E7107 (*29*) followed by classification of splicing events as skipped exon (SE), retained intron (RI), mutually exclusive exon (MXE), alternative 5′ splice site (A5SS), and alternative 3′ splice site (A3SS). Cells treated with E7107 showed RI changes at 15 min followed by a subsequent spike in SE events at 30 min, 1 hour, and 24 hours of treatment (Fig. 3A, fig. S3A, and table S2). These splicing alterations were drug dose dependent (fig. S3B) and had similarities to splicing events induced by *SF3B1* silencing using two independent shRNAs (*shSF3B1*) (fig. S3C and table S3). Similar to E7107 treatment, we identified SE as the main splicing change upon silencing of *SF3B1* (fig. S3, C to E). The commonly alternatively spliced transcripts between SF3B1 inhibition and silencing, which represent the most related SF3B1 targets, are enriched in DNA damage response (DDR) pathways (Fig. 3, B and C). Treatment with another clinically used SF3B1 inhibitor, H3B-8800, also showed similarities in the splicing changes with *shSF3B1* (fig. S3F), and commonly affected transcripts were enriched in DNA damage, cell cycle, and repair signatures (fig. S3G). Splicing alternation genes showed a wide overlapping between SF3B1 inhibition, *SF3B1* silencing, and P5091 treatment. The commonly changed transcripts are enriched in protein deacylation, G_2_-M phase transition, and DDR pathway (fig. S3, G and H). We also found that E7107 treatment (or *shSF3B1* expression) causes the use of a cryptic 3′ splice site approximately 64 nucleotides (nt) upstream of the canonical 3′ splice site [fig. S3, K and L, blue line; 64 nt (log_2_64 = 6)]. In contrast, cells with mutant *SF3B1* present with a cryptic 3′ splice site 15 nt upstream of the canonical 3′ splice site, fewer uridines, and more purine residues (*8*, *44*), suggesting differential impact yielded by *SF3B1* silencing and *SF3B1* mutation on A3SS splicing. These data demonstrate that SF3B1 silencing or inhibition leads to widespread exon skipping and cryptic 3′ splice site alterations.

To characterize the effect of SF3B1 inhibition on transcription, we performed κ-means clustering analysis of transcriptome changes upon *SF3B1* inhibition and identified both positive and negative impact on transcription in an E7107 dose-dependent manner (Fig. 3D and table S4). RNA metabolic processes were enriched in the positively regulated gene cluster (Fig. 3E). In contrast, the DDR-related pathways were enriched in the negatively affected cluster (Fig. 3E). The most significantly affected gene families upon SF3B1 inhibition were the DDR, with 44 affected transcripts, including *FANCA*, *ATM*, *ATR*, *RAD51*, and *CHEK2*, suggesting a potential role for SF3B1 in DDR regulation (Fig. 3F). *SF3B1* silencing led to similar conclusions (fig. S4, A to F, and table S5), with several overlapping transcripts up- and down-regulated between E7107 and *shSF3B1* (fig. S4, G and H). Conclusively, the expression of DDR transcripts is commonly impaired by both E7107 treatment and *SF3B1* silencing (fig. S4I).

The above findings suggest that SF3B1 might affect transcription level. Our analysis identified a notable number of negatively regulated transcripts that also exhibited an altered splicing pattern upon E7107 treatment (Fig. 3G). Past studies have shown that transcription and splicing are functionally intertwined with regard to both degradation of transcripts with premature termination codon via NMD after aberrant splicing caused by SF3B1 mutations or inhibition (*8*, *45*, *46*) and a potential role of SF3B1 and other splicing factors in active transcription (*14*–*16*, *47*). To explore the role of SF3B1 inhibition in transcript degradation via NMD, we assessed gene expression and splicing changes upon treatment with E7107 and an NMD inhibitor (NMDi) to block the interaction between SMG5 and the main adenosine triphosphatase (ATPase) and helicase involved in NMD, up-frameshift suppressor 1 homolog (UPF1) (*48*, *49*), and compared these effects with those of single-agent treatment with E7107. We observed a significant rescue of E7107-mediated gene expression down-regulation upon NMD inhibition (1722 of 4535 transcripts were rescued; Fig. 3H). Enrichment analysis showed that the expression of DDR gene family members, including *CHEK2*, *RAD51*, and *FANCD2*, was rescued upon NMDi treatment (Fig. 3, H and I), suggesting that SF3B1 inhibition reduces the levels of DDR transcripts partially via NMD degradation.

**Fig. 4.**
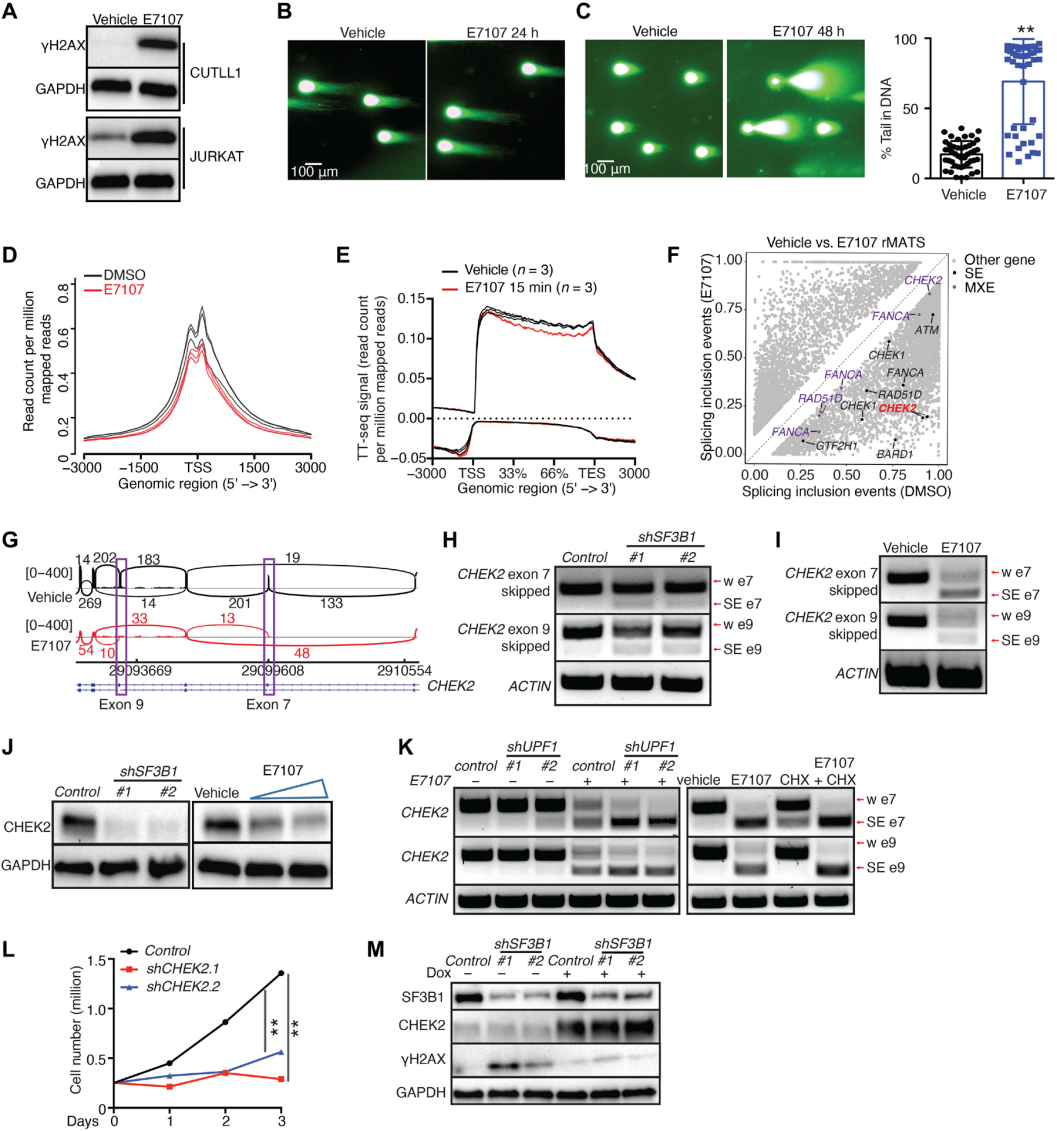
SF3B1 activity is critical for CHEK2 levels and DNA damage response. (**A**) γH2AX level in CUTLL1 and JURKAT cells treated with 3 nM E7107 for 24 hours. (**B** and **C**) Representative comet assay photos from CUTLL1 cells treated with 3 nM E7107 for 24 or 48 hours [quantification of (C) in the right panel, *n* = 45]. **P ≤ 0.01. (**D**) Metagene analysis of MapR signal around the transcriptional start site in 3 nM E7107 treatment for 24 hours in CUTLL1. DMSO, dimethyl sulfoxide. (**E**) Metagene analysis of normalized transient transcriptome sequencing (TT-seq) reads for protein-coding transcripts (±3-kb area, 3 nM E7107, 15 min). (**F**) Scatterplot of splicing changes in E7107 versus vehicle (FDR < 0.05 and PSI > 0.1). (**G**) Sashimi plots representing exon-exon junctions for *CHEK2* upon treatment of CUTLL1 cells with E7107 (3 nM, 24 hours). (**H** and **I**) PCR for the detection of *CHEK2* exon 7/9 skipping in *shSF3B1* CUTLL1 cells (H) or upon treatment with 3 nM E7107 [24 hours, (I)]. (**J**) CHEK2 protein levels in *shSF3B1* CUTLL1 cells or upon treatment with E7107 (1.5 and 3 nM) for 24 hours. (**K**) PCR for the detection of CHEK2 exon 7/9 skipping in *shUPF1* CUTLL1 cells treated with E7107 (3 nM, 24 hours) and CUTLL1 cells treated with 3 nM E7107 or 3 nM E7107 + CHX (50 μg/ml) for 8 hours (w e, with exon). (**L**) Growth of *shCHEK2.1*- and *shCHEK2.2*-expressing CUTLL1 cells (*n* = 3). (**M**) CHEK2 protein levels in *shSF3B1* CUTLL1 cells treated with doxycycline (Dox; 1.5 μg/ml, 48 hours) to induce ectopic expression of CHEK2.

Our findings suggest a role for SF3B1 in controlling the levels of DDR transcripts and led us to hypothesize that SF3B1 is implicated in the DDR pathway. We performed comet assay upon SF3B1 inhibition or silencing to determine the effect of SF3B1 on DNA damage. The levels of histone H2AX phosphorylation (γH2AX), a marker of double-strand breaks (DSBs), as well as the comet tail length, a surrogate of DNA damage, were both significantly increased upon E7107 treatment or *SF3B1* silencing (Fig. 4, A to C, and fig. S5, A to D). These findings suggest that SF3B1 silencing or inhibition causes a significant accumulation of DNA damage in T-ALL cells.

In the past decade, mutations in *SF3B1*, and other splicing factor genes, have been shown to compromise genome integrity, partially via accumulation of R-loops (*10*, *11*, *50*–*54*). R-loops consist of DNA/RNA hybrids and a single DNA strand, which may interfere with DNA replication, repair, and transcription; they form temporarily at sites of active transcription and can induce DSBs and DNA damage, if left unresolved, thus pausing a risk for DNA integrity, chromatin structure, and cell proliferation (*55*–*58*). To analyze R-loops upon SF3B1 inhibition, we used the MapR method, which uses the catalytically mutant ribonuclease (RNase) H to guide micrococcal nuclease to R-loops without cleaving the RNA strand; the R-loops were subsequently isolated and characterized by high-throughput sequencing (*59*). Our studies show that *SF3B1* inhibition over a period of 24 hours leads to changes in R-loop distribution in the genome and an overall reduction of R-loops (12,568 peaks in E7107 condition compared to 17,303 peaks in vehicle; Fig. 4D and fig. S5E). This finding suggests that changes in R-loops cannot explain the DDR phenotype observed in 24 hours after treatment, and the two phenomena are temporally and spatially uncoupled. Because of the suggested co-occurrence of transcription and splicing and the effect of SF3B1 inhibition on R-loop formation, we next sought out to characterize the impact of SF3B1 inhibition on active transcription by mapping nascent transcripts and performing transient transcriptome sequencing (TT-seq) (*60*), as a metric of active transcription, upon 15 min of E7107 drug treatment. Our analysis showed that splicing inhibition led to impaired transcriptional elongation (Fig. 4E) upon 15 min of E7107 treatment, and the effect was potentiated after 24 hours of E7107 treatment (fig. S5F). Impaired transcription might be the reason behind the reduction in the number of R-loops observed. In addition, the transcripts affected present with R-loop changes, suggesting a connection between SF3B1-mediated decrease in nascent transcription and a decrease in R-loops.

### CHEK2 is a critical SF3B1 target in T-ALL

To investigate the role of SF3B1 inhibition in DNA damage, we analyzed our sequencing data upon E7107 treatment for potential alterations in the splicing pattern of DDR-related transcripts. We identified changes in the splicing of general transcription factor II H subunit 1 and 2c (*GTF2H1* and *GTF2H2C*), Fanconi anemia group a and g (*FANCA* and *FANCG*), and checkpoint kinase 1 and 2 (*CHEK1* and *CHEK2*) (Fig. 4F and fig. S5G). The cell cycle–related transcript *CHEK2*, coding for CHEK2 kinase domain, is the DDR transcript presenting with the highest PSI (percent spliced in) score upon E7107 treatment for exon skipping (exons 7 and 9, PSI SE_7_ = 0.719, PSI SE_9_ = 0.739; Fig. 4G). The signaling axis driven by the kinases ATM and CHEK2 is considered the principal mediator of the DSB-repair pathway (*61*). *ATM* transcript is also affected via SE phenomena, albeit to a lower extent compared to CHEK2 (PSI SE = 0.57; Fig. 4F), suggesting that CHEK2 is the main pathway component affected via splicing upon SF3B1 inhibition.

Although identified *CHEK2* mutations suggested that CHEK2 is a candidate tumor suppressor in various types of cancer, such as breast cancer (*62*, *63*), recent evidence showed that *CHEK2* might also act as an oncogene shaping the response to chemotherapy (*64*, *65*) and checkpoint kinase inhibitors have shown promise as therapeutic agents (*66*, *67*). We confirmed exon skipping in the *CHEK2* transcript upon inhibition or silencing of *SF3B1* via polymerase chain reaction (PCR) (Fig. 4, H and I, and fig. S5H). Our additional analysis showed a marked increase of *CHEK2* mRNA expression in T-ALL cells compared to T cells (fig. S5I). *CHEK2* codes for a serine/threonine protein kinase regulating G_2_-M phase checkpoint in response to DNA damage (*68*). Skipping of exon 7 or 9 causes a reduction in CHEK2 transcript and protein levels potentially via a premature termination codon and transcript degradation via the NMD pathway (Fig. 4J and fig. S5, J to N). To further evaluate potential regulation of *CHEK2* via NMD, we silenced the main NMD component, the ATPase, and helicase *UPF1* (*69*, *70*) or treated the cells with CHX, which blocks NMD (fig. S5O). Both *shUPF1* and CHX treatment partially restored the levels of *CHEK2* transcripts with SE 7 or SE 9 (Fig. 4K), suggesting potential regulation of *CHEK2* via NMD, in line with our transcriptome studies previously presented in Fig. 3I.

To study the functional role of CHEK2 in T-ALL, we silenced *CHEK2* (*shCHEK2*) to observe an increase in DNA damage and apoptosis (fig. S6, A and B) and a decrease in proliferation, demonstrated via a G_2_-M arrest (Fig. 4L and fig. S6C) (*71*). The use of the CHEK2 inhibitor (BML277) led to a decrease in cell growth, apoptosis, and G_2_-M arrest similar to *shCHEK2* (fig. S5, D to F). Inducible ectopic expression of CHEK2 partially rescued the SF3B1-caused DNA damage (Fig. 4M), suggesting that *CHEK2* is a critical SF3B1 target. Together, our results suggest that blocking SF3B1 leads to *CHEK2* exon 9 skipping, which ultimately results in G_2_-M arrest and apoptosis.

### A splicing-based combination drug approach to target T cell leukemia cells

**Fig. 5.**
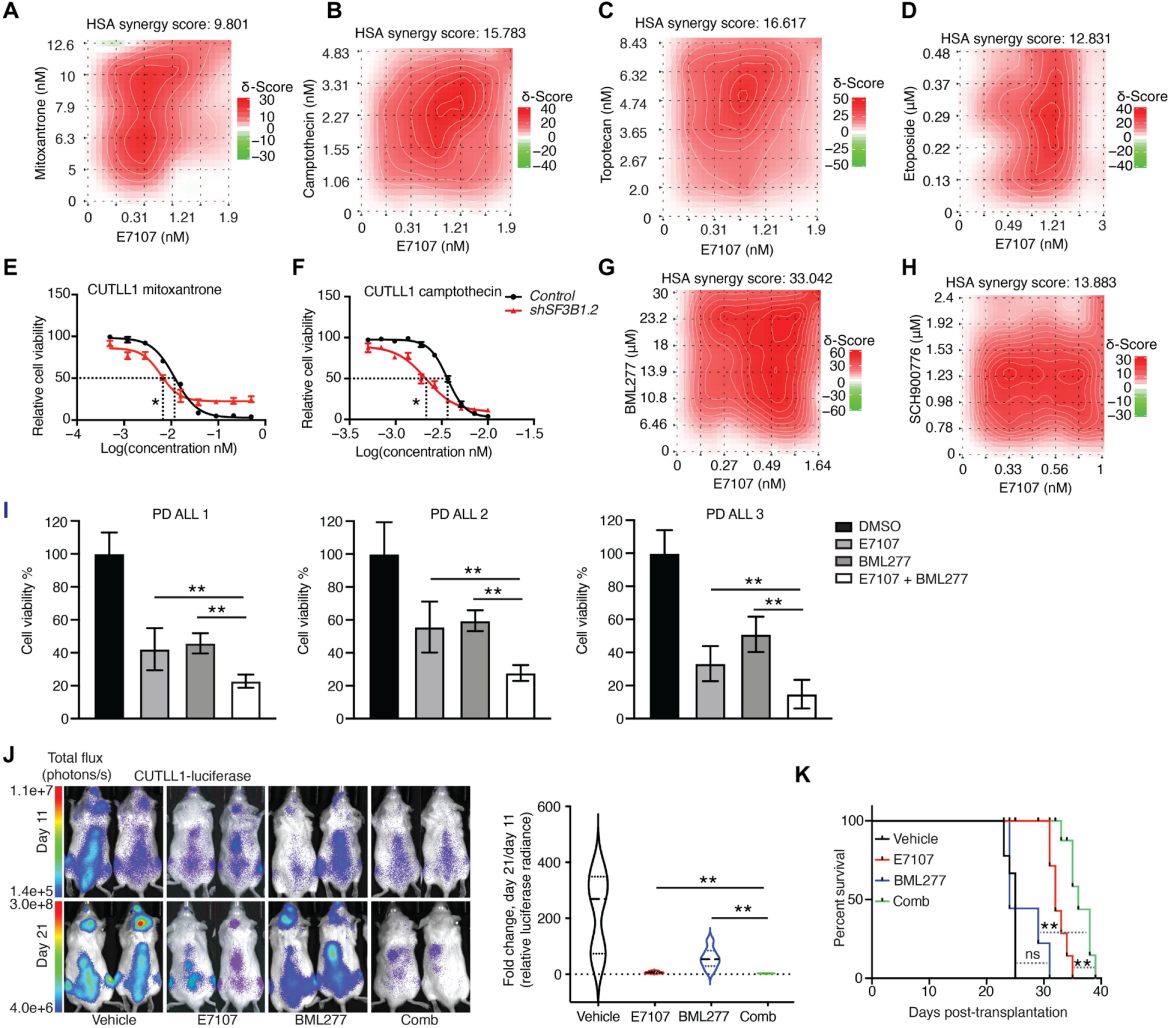
A splicing-based combinatorial drug approach in acute lymphoblastic leukemia. (**A** to **D**) Synergy heatmaps representing combination treatments of E7107 with mitoxantrone, camptothecin, topotecan, and etoposide (CUTLL1, 3 days). HSA, Highest Single Agent. Red color denotes drug synergy. (**E** and **F**) IC_50_ curves upon treatment of *control* and *shSF3B1#2* CUTLL1 cells with mitoxantrone and camptothecin. (**G** and **H**) Synergy heatmaps representing combination treatments of E7107 with BML277 or SCH900776 (CUTLL1, 3 days). Red color denotes drug synergy. (**I**) Representative cell viability upon the treatment of 2 nM E7107, 50 μM BML277, or 2 nM E7107 + 50 μM BML277 in patient samples for 24 hours. (**J**) Luciferase-expressing CUTLL1 cells were injected into immunocompromised mice via tail vein coupled to mouse treated with E7107 (at 5 mg/kg per day), BML277 (at 1 mg/kg per day), or E7107 and BML277 (“Comb”). Leukemic burden was assessed via blast detection in mouse body using bioluminescence and in vivo imaging system equipment twice per week. Images from luminescence analysis for representative mice on days 11 and 21 (left) and luminescence intensity fold change between days 11 and 21 are shown [right: vehicle (*n* = 5), E7107 (*n* = 7), BML277 (*n* = 7), or E7107 and BML277 (*n* = 7*)*]. **P ≤ 0.01. (**K**) Mouse survival analysis from (I) [vehicle (*n* = 5), E7107 (*n* = 7), BML277 (*n* = 7), or E7107 and BML277 (*n* = 7)].

Resistance to DNA damaging chemotherapy is a main unmet need in T-ALL. Driven by the high protein levels of SF3B1 in HR T-ALL samples and E7107-inflicted alterations in DDR, we used combinations of splicing factor inhibitors and chemotherapeutic drugs such as topoisomerase I inhibitors, topoisomerase II inhibitors, and mitoxantrone as single-agent treatments or in combination with E7107 to assess growth inhibition (Fig. 5, A to D, and fig. S7, A to D). E7107 synergized with chemotherapy agents, except doxorubicin, to block T-ALL growth. Treatment with the clinical splicing inhibitor H3B-8800 produced nearly identical results (fig. S7, E to N). *SF3B1* silencing rendered the T-ALL cell lines more sensitive to chemotherapeutic drugs than the control cells, confirming the impact of SF3B1 on the chemotherapeutic response (Fig. 5, E and F, and fig. S7, O and P). The increased γH2AX levels upon combination drug treatment further suggested synergy between SF3B1 silencing and chemotherapy in T-ALL (fig. S7Q).

We then tested the combination of E7107 and a CHEK2 inhibitor (BML277) (*72*, *73*) in T-ALL cell lines and found high synergy scores between the two compounds (Fig. 5G). SCH900776-induced inhibition of CHEK1, a kinase structurally similar to CHEK2 (*74*), also showed synergy with E7107 (Fig. 5H). E7107 and CHEK2 combined had the highest synergy score among all small molecules tested (Fig. 5H). To test this drug combination in a more physiologically relevant setting, we used treatment of patient samples with E7107 and CHEK2 inhibitors. We observed a strong inhibitory effect on patient sample viability (Fig. 5I) similarly to human cell lines previously tested. This prompted us to further test this drug combination in a preclinical T-ALL model. We transplanted luciferase-expressing CUTLL1 cells into immunocompromised mice and then treated the mice with E7107 and BML277 as single drugs or in combination upon tumor detection. Mice treated with the drug combination showed a further tumor regression compared with single treatment, associated with a prolonged mouse survival (Fig. 5, J and K). Our analysis failed to detect notable toxicity associated with body or organ weight, blood populations, goblet cell metaplasia, or other gastrointestinal toxicity such as the one previously demonstrated for gamma secretase inhibitor drugs used to block NOTCH1 activity in T-ALL therapy (fig. S8, A to E, and table S1) (*34*).

Overall, our data indicate that splicing inhibition has a strong synergistic effect with epigenetic and transcriptional inhibition and chemotherapy drugs against T-ALL cell growth and no significant toxicity, and demonstrate the therapeutic potential of targeting the spliceosome in T-ALL.

## DISCUSSION

Although SF3B1 is one of the most commonly mutated splicing factors in adult cancers, pediatric cancers, such as T-ALL, exhibit very few splicing mutations (*26*). However, T-ALL exhibits aberrant splicing and sensitivity to SF3B1 inhibition. Our present study identifies a previously unidentified regulatory mechanism of the U2 component SF3B1 in pediatric leukemia via USP7 activity and protection against degradation. Further research is warranted to identify SF3B1 degradation pathways and reveal the potential interaction between ubiquitination and other posttranslational modifications in controlling SF3B1 levels and activity.

Driven by our observation that SF3B1 inhibition leads to a reduction of R-loops in the genome, we mapped changes in the nascent transcriptome upon SF3B1 inhibition in leukemia. Our molecular findings, combined with the results of biochemical studies, showed that SF3B1 plays a critical role in the transcription of oncogenes as well as DDR transcripts. Considering the importance of splicing regulation in tumors expressing high levels of the transcriptional amplifier MYC (*20*), we think that our findings might provide an explanation for this dependency via the participation of SF3B1 in active transcription. SF3B1 levels are elevated in HR tumors, and the new generation of splicing inhibitors has exhibited minimal toxicity in the clinic (*75*), suggesting that high SF3B1 levels are associated with therapeutic resistance and might have important implications in therapeutic response.

The use of splicing inhibitors in the clinic as single agents has shown promise, and the new-generation compound H3B-8800 was shown to be sufficiently safe in a phase 1 study (*30*, *75*). Nevertheless, patients with myeloid malignancies treated with H3B-8800 showed hematological improvement but no partial or complete responses. In our study, we demonstrated the efficacy of SF3B1 inhibitors in patient samples, and mouse and xenograft preclinical models of T-ALL. We further identified that SF3B1 inhibition affects a novel exon-skipping event and ultimately the levels of *CHEK2* transcript, and we also showcase the importance of CHEK2—the most significantly impaired DDR transcript upon SF3B1 inhibition—as a critical regulator of DDR downstream of SF3B1. Previous studies have demonstrated the increase in R-loop formation in cells with mutant U2AF1 and sensitization to ATR kinase inhibitors upon SF3B1 inhibition (*76*). Further investigation is needed to disentangle the effect of SF3B1 inhibition in R-loop formation and DDR in spliceosome wild-type and mutant tumors.

Our studies show that CHEK2 is a critical SF3B1 target, as ectopic expression of CHEK2 demonstrates high synergy of SF3B1 inhibitors with CHEK2 inhibitors as well as chemotherapeutic drugs, and propose that the SF3B1 inhibitor E7107 is a lead compound that sensitizes cancer cells to chemotherapy. Given the fact that SF3B1 and CHEK2 inhibition and their combination present with very low toxicity against hematopoietic progenitor cells and in preclinical models [our current study and (*26*)], splicing-based drug combinations comprise a potential strategy for improving the efficacy of treatment and overcoming resistance in T-ALL with no significant associated toxicity.

## METHODS

### Cell lines and primary cells

The human T-ALL cell lines CUTLL1 (gift from A. Ferrando, Columbia University) and JURKAT [American Type Culture Collection (ATCC), Manassas, VA, #CCL-119] were cultured in RPMI 1640 medium supplemented with 10% heat-inactivated fetal bovine serum (FBS) (Sigma-Aldrich, St. Louis, MO), 1% penicillin/streptomycin (Gibco, Thermo Fisher Scientific, Hampton, NH), and 1% GlutaMAX (Gibco, Thermo Fisher Scientific). 293T cells (ATCC, #CRL-11268) were cultured in Dulbecco’s modified Eagle’s medium supplemented with 10% heat-inactivated FBS, 1% penicillin/streptomycin, and 1% GlutaMAX. The cells were periodically tested for the presence of mycoplasma using the Lonza Walkersville MycoAlert Mycoplasma Detection Kit. The cell lines had been authenticated using short-tandem repeat profiling (JURKAT) or using PCR to detect the TCRb-NOTCH1 translocation (TCRBJ2S4CUTLL1F: 5′-GGACCCGGCTCTCAGTGCT-3′, NOTCH1CUTTL1R: 5′-TCCCGCCCTCCAAAATAAGG-3′). Human CD3^+^, CD8^+^, and CD4^+^ T cells were purchased from AllCells.com (Alameda, CA) or from Astarte Biologics (Bothell, WA). Primary human samples were collected by collaborating institutions with informed consent and analyzed under the supervision of the Institutional Review Board of Padova University, the Associazone Italiana Ematologia Oncologia Pediatrica, and the Berlin-Frankfurt-Münster (AIEOP-BFM) ALL 2000/2006 pediatric clinical trials. Informed consent to use leftover material for research purposes was obtained from the patients at trial entry in accordance with the Declaration of Helsinki. Patient cells were cultured with α-MEM medium [10% human heat-inactivated AB+ serum, 10% FBS, 0.5% penicillin/streptomycin, 1% glutamine, human interleukin-7 (10 ng/ml), human stem cell factor (50 ng/ml), human FLT3 ligand (20 ng/ml), and 20 nM insulin]. Cell viability was tested using alamarBlue.

### Cell transfection, virus production, and cell infection

293T cells that reach up to 80% confluency were transfected by using jetPrime reagent following the recommended protocol (Polyplus, France). Virus was collected for the subsequent experiment as required after 48 hours. The following shRNAs (Sigma-Aldrich, MISSION system) were used: *shSF3B1#1* (TRCN0000320566), *shSF3B1#2* (TRCN0000350273), *shCHEK2#0* (#TRCN0000010209), *shCHEK2#1* (#TRCN0000010312), and *shCHEK2#2* (#TRCN0000010213). *shRNA* control: 5′-CCGGCAACAAGATGAAGAGCACCAACTCGAGTTGGTGCTCTTCATCTTGTTGTTTTT-3′. *shRNAs* of *UPF1* were purchased from Addgene (#136036 and #136037). Cells (0.5 million) were resuspended by using 2 ml of virus plus polybrene (10 μg/ml). The cells were spun down at 32°C, 2500 rpm for 2 hours, and resuspended with fresh medium. After 36 hours, cells were selected by antibiotic.

### Cell growth and viability assays, apoptosis, and cell cycle analysis

To study cell growth, 3000 cells per well were seeded using a microplate dispenser (MultiFlo, BioTek, Winooski, VT) in 384-well clear-bottom, black-wall plates (Corning, Corning, NY), and drugs were added using a Tecan D300e digital dispenser (Tecan, Männedorf, Switzerland). After 72-hour incubation, alamarBlue cell viability reagent (Thermo Fisher Scientific, Waltham, MA) was added and viability was quantified by measuring fluorescence in a plate reader (Tecan Infinite M1000 PRO, λ_ex_: 530 nm; λ_em_: 590 nm). Synergy score is evaluated by https://synergyfinder.fimm.fi/synergy/20200703210859780364/ (*77*). For apoptosis analysis, cells were stained with BD Phospholipid-binding protein, APC Annexin V (BD, 550474) according to the manufacturer’s protocol, except that cells were stained for 15 min at room temperature (RT). Flow cytometry was used for signal detection. For cell cycle analysis, cells were fixed in 100 μl of Fix and Perm Medium A (Life Technologies) for 15 min at RT, washed with 3 ml of phosphate-buffered saline (PBS), and incubated with 0.1% Triton X-100 in water, supplemented with 4′,6-diamidino-2-phenylindole (DAPI) (1 μg/ml) (Invitrogen, Carlsbad, CA) for 24 hours at 4°C. Flow cytometry was performed on LSR II (BD, Franklin Lakes, NJ), and analyses were performed using FlowJo software (Tree Star, Ashland, OR). Statistical analyses were performed using GraphPad Prism software (GraphPad Software, CA) using Student’s unpaired two-sided *t* test. For the IC_50_ of E7107 in patient samples, or patient samples that had passed through, mice (patient-derived xenografts) were treated with different concentrations of E7107 to evaluate the cell growth.

### Antibodies and reagents

The following antibodies were used for Western blotting or immunoprecipitation: anti-SF3B1 (Bethyl Laboratories, A300-996A-T, MBL International, D221-3), rabbit anti-cleaved Notch1 (Cell Signaling Technology, 4147S), rabbit anti–β-actin (Cell Signaling Technology, 4967S), rabbit anti-USP7 (Bethyl Laboratories, A300-033A), rabbit anti-GAPDH (glyceraldehyde-3-phosphate dehydrogenase) (Cell Signaling Technology, 2118S), rabbit anti-CHEK2 (Bethyl Laboratories, A300-618A), mouse immunoglobulin G2b (IgG2b) (MBL International, M077-3), P5091 (Selleck Chemicals, S7132), PR619 (Sigma-Aldrich, SML0430), b-AP15 (Selleck Chemicals, S4920), ML323 (Sigma-Aldrich, SML1177), BML277 (MedChem Express, HY-13946), BML277 (Sigma-Aldrich, C3742, in vivo), SCH900776 (MedChem Express, HY-15532), camptothecin (Selleck Chemicals, S1288), flavopiridol (Sigma-Aldrich, F3055), topotecan (Sigma-Aldrich, T2705), etoposide (Sigma-Aldrich, E1383), mitoxantrone (European pharmacopoeia reference standard, M2305000), doxorubicin (Sigma-Aldrich, 44583), and E7107 and H3B-8800 (H3 Biomedicine).

### Immunoprecipitation

Two hundred million T-ALL cells were collected. Cells were resuspended in 5 volumes of lysis buffer [50 mM tris (pH 7.5), 1 mM EDTA, 150 mM NaCl, 1% (v/v) Triton X-100, 1:100 protease inhibitor (Sigma-Aldrich, P8340), 1 mM NaV, and 1 mM NaF] containing 5 mM MgCl_2_ and benzonase (1 U per million cells) and incubated at 4°C for 30 min, rotating. Lysates were passed through a 25 ^1^/_2_-gauge needle/syringe five times and spun down at 4°C, 12,000 rpm, for 15 min to remove debris. Protein G magnetic beads were added to the lysates to decrease nonspecific binding and incubated at 4°C for 30 min, rotating. Precleared lysates were then incubated with the appropriate antibody-conjugated beads (5 mg of antibody per 100 million cells) at 4°C overnight, rotating. Beads were washed three times in high sodium chloride buffer [50 mM tris (pH 7.5), 1 mM EDTA, 300 mM NaCl, 1% (v/v) Triton X-100] and then washed three times with lysis buffer [50 mM tris (pH 7.5), 1 mM EDTA, 150 mM NaCl, 1% (v/v) Triton X-100] at 4°C for 5 min.

### RNA isolation, sequencing, and PCR

RNA was extracted from cell lines and patient samples using the Bio-Rad Total RNA Isolation Kit. Poly(A)-selected, unstranded Illumina libraries were generated using the TruSeq RNA Kit from Illumina. Library fragments were amplified with PCR, size-selected using AMPure XP beads to select for fragments between 200 and 500 base pairs (bp), and sequenced on Illumina NextSeq 500 in a paired-end run (2 × 76 bp) for a sequencing depth of about 80 million reads per sample.

The following splicing event primers were used: *CHEK2*-exon7-F: TGGTGCCTGTGGAGAGGTAA and *CHEK2*-exon7-R: TTGTCAAACAGCTCTCCCCC; *CHEK2*-exon9-F: GAAGGGGGAGAGCTGTTTGAC and *CHEK2*-exon9-R: GCCAAGTAGGTGGGGGTTC; *FANCA*-F: CTGGAGACCCTTGCACCTTC and *FANCA*-R: GGCATTTCGTCTGGCACTTG; *FANCG*-F: TATCCAGCGGAGCCTAGAGAG and *FANCG*-R: TGTGTACACCTGGACCAACA; *GTF2H2C*-F: GTTTCCGGCTGAGAGTCCTT and *GTF2H2C*-R: TGGCAGGTCATATCCACAGC; β*-actin*-F: CATGTACGTTGCTATCCAGGC and β-*actin*-R: CTCCTTAATGTCACGCACGAT.

The following reverse transcription PCR (RT-PCR) primers were used: *CHEK2*-F: TCTCGGGAGTCGGATGTTGAG and *CHEK2*-R: CCTGAGTGGACACTGTCTCTAA; *SF3B1*-F1: GTGGGCCTCGATTCTACAGG and *SF3B1*-R1: GATGTCACGTATCCAGCAAATCT; *SF3B1*-F2: TTTGCTGGATACGTGACATCAA and *SF3B1*-R2: CCGGTCTGCAATCTTTGGAGG.

### Single-cell gel electrophoresis (comet assay)

CUTLL1 and JURKAT cells were infected with *control* or *shSF3B1* and selected as described before. CUTLL1 and JURKAT cells were treated with 3 nM E7107 for 24 or 48 hours. Cells (0.1 million) were used for single-cell gel electrophoresis by following the instructions from Trevigen (catalog no. 4250-050-K). Images were acquired with Nikon Eclipse Ts2. The comets were quantified by using plugin extension of ImageJ called OpenComet.

### Immunoblots and RPPA

To make total cell extracts, up to 3 million cells were collected and resuspended in 40 μl of radioimmunoprecipitation assay buffer [50 mM tris-HCl (pH 8.0), 150 mM NaCl, 1% NP-40/IGEPAL, 0.5% sodium deoxycholate, 0.1% SDS, 1:100 protease inhibitor (Sigma-Aldrich, P8340), 1 mM NaV, and 1 mM NaF in H_2_O] per 1 million cells. Cells were lysed on ice for 20 min and spun down at 4°C, maximum speed, for 10 min to remove debris. The protein concentration was determined by using the BCA Protein Assay Kit (Thermo Fisher Scientific, 23225). Then, the protein samples were separated by SDS–polyacrylamide gel electrophoresis using 4 to 15% tris-glycine polyacrylamide gels (Bio-Rad) and transferred to polyvinylidene difluoride membranes (Millipore), which were blocked with 5% fat-free milk for 60 min. Then, the membranes were incubated with primary antibodies at 4°C overnight followed by the corresponding secondary antibodies. Reverse-phase protein array (RPPA) was performed as described previously (*26*).

### Ubiquitination assays

293T cells were transfected with hemagglutinin (HA)/MYC-ubiquitin and Flag-SF3B1, and the cells were treated with either dimethyl sulfoxide (DMSO) or 10 μM P5091 USP7 inhibitor after 24 hours of the transfection for another 24 hours. The cells were lysed with in SDS lysis buffer [20 mM tris-HCl (pH 7.4), 150 mM NaCl, 1 mM EDTA, and 1% SDS] mixed with a protease inhibitor cocktail (Roche, 04693132001). After denaturation by heating, the lysate was diluted 10-fold with lysis buffer [20 mM tris-HCl (pH 7.4), 150 mM NaCl, 1 mM EDTA, and 1% Triton X-100], sonicated, centrifuged at 15,000 rpm for 15 min at 4°C, and then incubated with protein G magnetic beads and the desired antibodies overnight. The final immune complexes were harvested after wash with high sodium chloride lysis buffer [20 mM tris-HCl (pH 7.4), 500 mM NaCl, 1 mM EDTA, and 1% Triton X-100] for three times and washed with low sodium chloride lysis buffer [20 mM tris-HCl (pH 7.4), 150 mM NaCl, 1 mM EDTA, and 1% Triton X-100] for three times, 5 min for each wash.

### Intravenous xenograft studies and drug treatment in vivo study

All mice were housed in a barrier facility, and procedures were performed as approved by the Northwestern University Institutional Animal Care guidelines and related mouse protocols under P.N. For CUTLL1-luciferase *contro*l and *shSF3B1* T-ALL intravenous studies, 1 million cells in 100 μl of PBS were injected into the eye vein of 8-week-old NOD.Cg-Prkdcscid female mice (The Jackson Laboratory, Portage, MI, #005557). Animals were monitored by IVIS (in vivo imaging system) twice per week for luciferase signal detection. IVIS images were taken using an IVIS Spectrum in vivo imaging system (PerkinElmer). For CUTLL1-luciferase T-ALL E7107 in vivo study, 0.25 million cells in 100 μl of PBS were injected into the tail vein of 8-week-old NOD.cg-Prkdcscid female mice (The Jackson Laboratory, Portage, MI, #005557). E7107 was dissolved in vehicle (10% ethanol and 5% Tween 80 in sterile PBS) and administered via intravenous tail vein injection at 5 mg/kg per day starting on day 10 after transplantation. Eight doses of E7107 were administered (five consecutive days, followed by 2-day rest, coupled to three consecutive days of treatment). Animals were monitored by IVIS twice per week for luciferase signal detection. IVIS images were taken using an IVIS Spectrum in vivo imaging system (PerkinElmer). For the combination treatment in vivo study, 0.25 million cells in 100 μl of PBS were injected into the tail vein of 8-week-old NOD.cg-Prkdcscid female mice (The Jackson Laboratory, Portage, MI, #005557). E7107 was dissolved in 10% ethanol and 5% Tween 80 in sterile PBS and administered via intravenous tail vein injection at 5 mg/kg per day starting on day 10 after transplantation. BML277 was dissolved in 10% ethanol (in sterile PBS) and administered via intraperitoneal injection at 1 mg/kg per day starting on day 10 after transplantation. Nine doses of E7107 were administered (six consecutive days, followed by 2-day rest, coupled to three consecutive days of treatment). BML277 was administered over a period of 17 days (every other day for first 5 doses coupled to single doses for 12 consecutive doses). Animals were monitored for luciferase signal detection using an IVIS imaging system (PerkinElmer) twice per week.

For the NOTCH1-ΔE-Cherry^+^ retroviral T-ALL model, c-kit hematopoietic progenitor cells from the bone marrow of the B6;129-Myctm1Slek/J mouse were isolated and infected with Notch-ΔE-cherry–expressing retrovirus coupled to transplantation of 85,000 NOTCH1/Cherry^+^ cells into animals. A total of 200,000 tumor cells isolated from the spleen of this primary transplantation model were transplanted into immunocompromised mice coupled to mouse treatment with eight doses of E7107 (five consecutive days, followed by 2-day rest, coupled to three consecutive days of treatment) administered via intravenous tail vein injection at 5 mg/kg per day starting on day 10 after transplantation.

For the mice toxicity assay, 8-week-old NOD.cg-Prkdcscid female mice (The Jackson Laboratory, Portage, MI, #005557) were separated into four groups (three mice per group) without transplantation. The mice received the same dose and times as the transplantation experiment groups. Upon death of the last mouse of the efficacy group, the mice were euthanized and weighted. Retro-orbital bleeding was performed to conduct the complete blood count test. Carbon dioxide euthanasia was then performed before dissecting the mice. The liver and spleen were weighed. The stomach, esophagus, jejunum, and ileum were harvested, washed with PBS, and placed in formalin (Sigma-Aldrich, #HT501128). The tissues were processed at the Mouse Histology and Phenotyping Lab at Northwestern University in a Sakura Tissue-Tek VIP6 processor and embedded using a Leica EG1160 embedding center. Four-micrometer sections of the tissues were stained with hematoxylin and eosin on an automated Leica Autostainer XL. Periodic acid–Schiff stain was performed manually. Briefly, the slides were placed in 0.5% periodic acid solution for 5 min after deparaffinization and hydration. After washing in distilled water, the slides were placed in Schiff reagent for 15 min and then washed with 0.55% potassium metabisulfite. The slides were counterstained in hematoxylin with acetic acid after washing in water and dehydrated and mounted with synthetic resin. The stained slides were blindly reviewed by a pathologist first and then re-reviewed by the pathologist after unblinding the treatment groups. Photos were acquired at ×200 magnification using an Olympus BX53 microscope, an Olympus U-TVO.63XC camera, and the Olympus cellSens Standard v2.2 software.

### Analysis of data from publicly available databases

#### Broad Institute CCLE database

The CCLE (www.broadinstitute.org/ccle) database was used to retrieve the mRNA expression of U2 components in various cancer cell lines, and the data are illustrated as box plots.

#### Bioinformatics analysis

RNA sequencing data analysis, such as gene expression changes, replicate multivariate analysis of transcript splicing (rMATS), exon count tables, isoform prediction and differential expression, and enrichment analysis, was exactly as described in our previous study (*26*). Enriched Kyoto Encyclopedia of Genes and Genomes (KEGG) and Reactome pathway terms were identified using EnrichR and plotted in ggplot2 package in R. Cryptic sites and density plots (*44*): For each pair of A3SS, the distance between was calculated as the difference of its chromosomal coordinates, and the log_2_ of the difference was plotted using density plots in the ggplot2 package in R. DDR gene list was retrieved from http://repairtoire.genesilico.pl/proteins/ and www.mdanderson.org/documents/Labs/Wood-Laboratory/human-dna-repair-genes.html.

#### TT-seq data analysis

CUTLL1 cells (10 million per group) were treated with 3 nM E7107 or vehicle for 15 min or 24 hours and labeled with 500 μM 4-thiouridine for 10 min. Cells were harvested, and RNA was extracted using TRIzol. Total RNA was fragmented and then biotinylated using MTSEA-biotin, followed by enrichment using streptavidin magnetic beads before rRNA depletion (NEBNext rRNA Depletion Kit, NEB #E6310L). Library was prepared using the NEBNext Ultra II Directional RNA Library Prep Kit (NEB, #E7645S).

Sequencing reads were mapped to the assembled reference genome (hg19/GRCh37.75) using the STAR aligner (v2.5.0c). Alignments were guided by a gene transfer format (GTF) file. The mean read insert sizes and their SDs were calculated using Picard tools (v.1.126) (http://broadinstitute.github.io/picard). The read count tables were generated using HTSeq (v0.6.0), normalized based on their library size factors using DEseq2, and differential expression analysis was performed. The read per million (RPM)–normalized BigWig files were generated using BEDTools (v2.17.0) and bedGraphToBigWig tool (v4). Two methods were used to compare the level of similarity among the samples and their replicates: principal components analysis and Euclidean distance–based sample clustering. ngs.plot (v2.47) was used to visualize the gene body profile plots for both strands and each strand separately, with 1% of the extreme values trimmed off. All the downstream statistical analyses and generating plots were performed in R environment (v3.1.1) (www.r-project.org/).

#### MapR data analysis

CUTLL1 cells (3 million per group) were treated with DMSO or 3 nM E7107 for 24 hours, and the lysates were treated with GST-RNaseH-MNase and GST-RNaseH mut-MNase as previously described (*59*). All of the reads from the sequencing experiment were mapped to the reference genome (hg19/GRCh37.75) using Bowtie2 (v2.2.4), and duplicate reads were removed using Picard tools (v.1.126) (http://broadinstitute.github.io/picard/). Low-quality mapped reads (MQ < 20) were removed from the analysis. The RPM-normalized BigWig files were generated using BEDTools (v.2.17.0) and the bedGraphToBigWig tool (v.4). Peak calling was performed using MACS (v1.4.2), and peak count tables were created using BEDTools. Differential peak analysis was performed using DESeq2. ChIPseeker (v1.8.0) R package was used for peak annotations. ngs.plot (v2.47) and ChIPseeker were used for transcriptional start site (TSS) visualizations and quality controls. Principal components analysis and Euclidean distance–based sample clustering were used for the comparison of the level of similarity among the samples and their replicates.

## Notes

View/request a protocol for this paper from *Bio-protocol*.
