# Supplementary material for: SF3B1 homeostasis is critical for survival and therapeutic response in T cell leukemia
Source: Sci Adv. 2022 Jan 21;8(3):eabj8357. doi: 10.1126/sciadv.abj8357 (PMC8782448; doi:10.1126/sciadv.abj8357)
Supplement: Supplementary file 1 — Figs. S1 to S8 [file sciadv.abj8357_sm.pdf]

Science Advances
AAAS

Supplementary Materials for

SF3B1 homeostasis is critical for survival and therapeutic response in T cell leukemia

Cuijuan Han, Alireza Khodadadi-Jamayran, Adam H. Lorch, Qi Jin, Valentina Serafin, Ping Zhu, Yuliya Politanska, Limin Sun, Blanca T. Gutierrez-Diaz, Marina V. Pryzhkova, Hiam Abdala-Valencia, Elizabeth Thomas Bartom, Barbara Buldini, Giuseppe Basso, Sadanandan E. Velu, Kavitha Sarma, Basil B. Mattamana, Byoung-Kyu Cho, Rebecca C. Obeng, Young Ah Goo, Philip W. Jordan, Aristotelis Tsirigos, Yalu Zhou, Panagiotis Ntziachristos*

*Corresponding author. Email: pntziachr@gmail.com; panagiotis.ntziachristos@ugent.be

Published 21 January 2022, *Sci. Adv.* **8**, eabj8357 (2022)
DOI: 10.1126/sciadv.abj8357

The PDF file includes:

Figs. S1 to S8

Other Supplementary Material for this manuscript includes the following:

Tables S1 to S5

Figure S1. Han et al., 2020

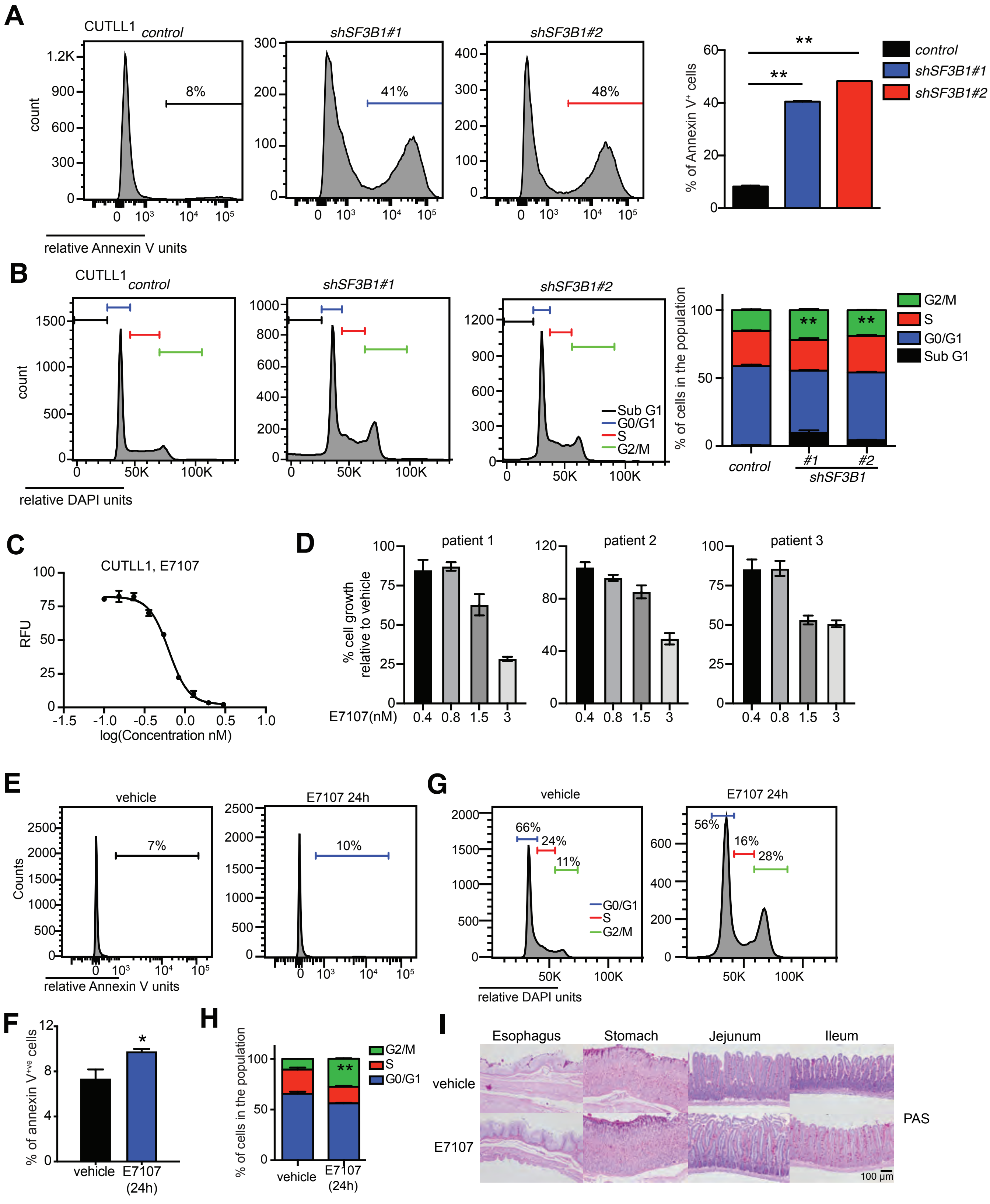

Supplementary Fig. 1. SF3B1 silencing or inhibition leads to apoptosis and G2/M arrest in T-ALL cells. A. Representative plots of annexin V staining levels assessed via flow-cytometry are shown for *control*, *shSF3B1#1* and *shSF3B1#2*-expressing CUTLL1 cells 48 h post-puromycin selection. Quantification of annexin V staining is shown (*n=3*, ** *P<0.01*; by two-tails unpaired Student's *t*-test). **B.** DAPI staining for overnight coupled to flow-cytometry analysis, for *control*, *shSF3B1#1* and *shSF3B1#2*-expressing CUTLL1 cells 48 h post-puromycin selection. Quantification of the cell cycle phases is shown in the right (*n=3*, ** *P<0.01;* by two-tails unpaired Student's *t*-test). **C.** Representative the IC50 curve upon treatment with E7107 in CUTLL1 cells. **D.** Cell growth upon treatment of patient-derived tumor with E7107. **E.** Annexin V staining upon treatment with vehicle or 3 nM E7107 for 24 h (CUTLL1). **F.** Representative graphs depicting the percentage of annexin V-positive cells in E (*n=3*, * *P<0.05*; ** *P<0.01;* by two-tails unpaired Student's *t*-test). **G.** DAPI staining for 3 nM E7107-treated CUTLL1 cells over periods of 24 h in CUTLL1 cells. **H.** Quantification of different cell cycle phases (data from G, *n=3*, ** *P<0.01;* by two-tails unpaired Student's *t*-test). **I.** Representative images of esophagus, stomach, jejunum and ileum (Periodic acid-Schiff (PAS) staining). 200x magnification is shown.

Figure S2. Han et al., 2020

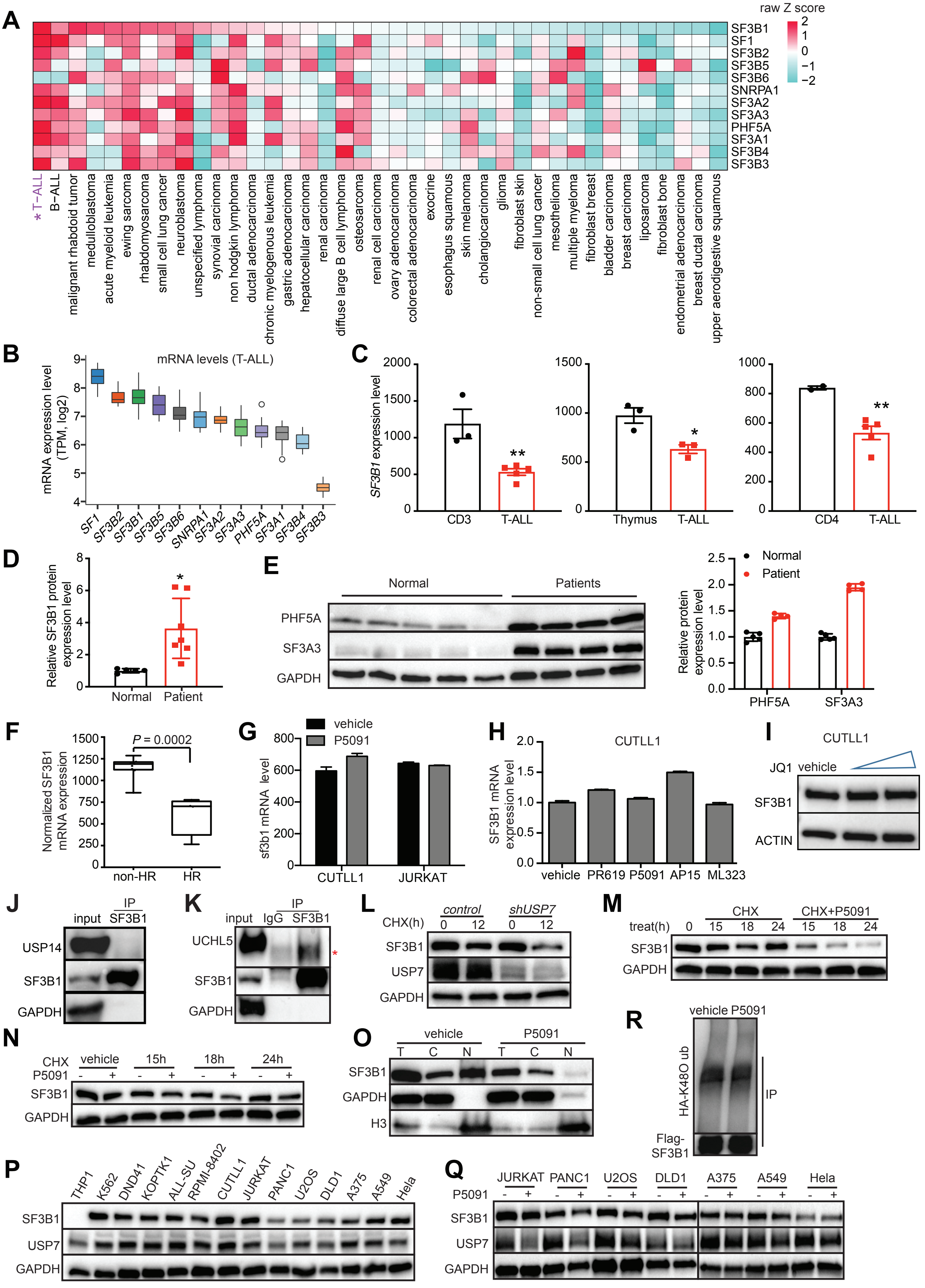

Supplementary Fig. 2. SF3B1 highly expressed in T-ALL cells and decreased upon USP7 inhibition. A. Heatmap representation of gene expression levels of U2 splicing complex components in cancer cell lines from the CCLE (Cancer Cell Line Encyclopedia) database. **B.** Gene expression analysis for U2 splicing complex components in T-ALL cell lines from the CCLE database. **C.** *SF3B1* mRNA expression level analysis in T-ALL vs. CD3+, CD4+ or thymocytes. RNA-Sequencing data from (*26*). **D.** Quantification of protein levels showed in the Fig 2B. **E.** Immunoblot showing PHF5A and SF3A3 protein level in patients versus T cells (left) and quantification analysis (right). **F.** *SF3B1* mRNA expression level in non-HR vs. HR T-ALL. **G.** *SF3B1* mRNA expression levels in vehicle vs. P5091 treatment for 24 h in RNA-seq database. **H.** RT-PCR showing *SF3B1* mRNA expression level upon treatment of CUTLL1 cells with 200 nM ML323, 10 μM PR619 and 2 μM b-AP15 for 24h. **I.** Immunoblot for SF3B1 levels in CUTLL1 cells treated with 0.5 μM or 1 μM JQ1 for 24h. **J, K.** Representative co-immunoprecipitation analysis for USP14 and SF3B1 or UCHL5 and SF3B1 (* non-specific band). **L.** Representative immunoblot of SF3B1 protein expression in control and *shUSP7* JURKAT cells upon treatment with 5μg/ml CHX for 12h. **M, N.** Representative immunoblot of SF3B1 protein expression upon treatment with 5μg/ml CHX or 5μg/ml CHX plus 10μM P5091 for different time points as indicated in RPMI-8402 cells (M) or JURKAT (N). **O.** Representative immunoblot of SF3B1 protein distribution in nuclear and cytoplasm upon treatment with 10 μM P5091 for 24 h in CUTLL1 cells. **P.** Representative immunoblot of SF3B1 protein expression in different cell lines. **Q.** Representative immunoblot of SF3B1 protein expression upon treatment with 10 μM P5091 (24 h) in different cells. **R.** Immunoprecipitation of flag-tagged SF3B1 coupled to immunoblot analysis for SF3B1 ubiquitination analysis in 293T cells expressing flag-SF3B1 and HA-K48O mutant ubiquitin construct (where only K48 can be ubiquitinated) treated with vehicle or P5091 USP7 inhibitor (10 μM, 24 h).

Figure S3. Han et al., 2020

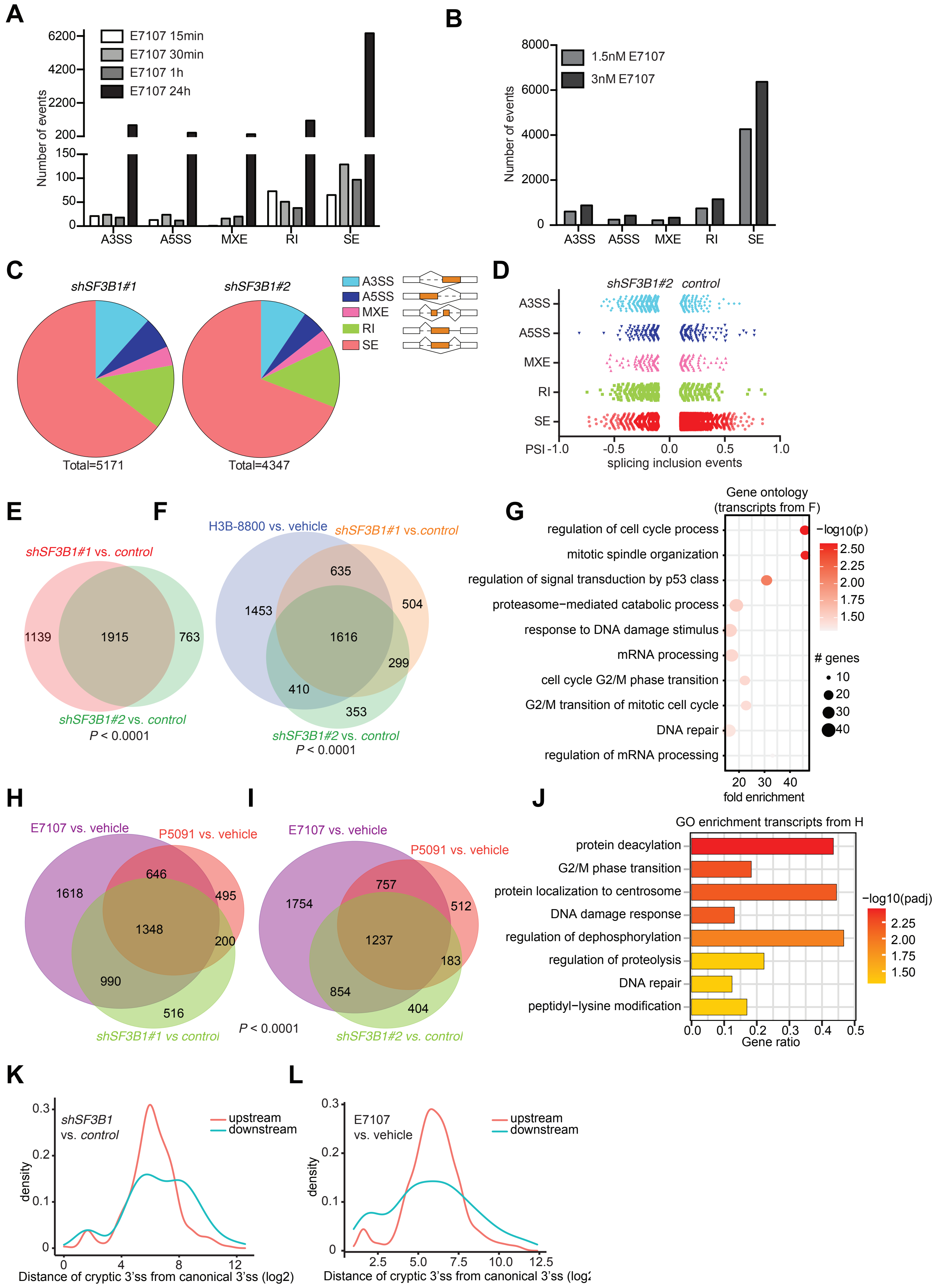

Supplementary Fig. 3. SF3B1 controls the levels of DDR splicing. A. 3nm E7107-induced splicing events in CUTLL1 cells in 15 min, 30 min, 1 h and 24 h of drug treatment. **B.** E7107-dose-associated splicing changes in CUTLL1 cells treated with 1.5 nM and 3 nM E7107 for 24 h. **C.** Total splicing events upon *SF3B1* silencing in CUTLL1 cells. The alternative splicing events are classified into 5 categories: SE, RI, MXE, A5SS and A3SS. **D.** Representation of E7107-induced splicing events changes in CUTLL1 cells (*FDR<0.05, PSI>0.1*). **E.** Overlap shows the common genes with alternative splicing events in two hairpins (right). Events with *FDR<0.05* and *PSI>0.1* are presented. Analysis shows 1915 common genes in all comparisons (*P<0.001*). **F.** Representation of overlapping transcripts presenting with splicing changes in control- vs. *shSF3B1#1*, control vs. *shSF3B1#2* and CUTLL1 cells upon treatment with H3B-8800 for 6 h vs. vehicle. Analysis shows 1616 common genes in all comparisons (*P<0.001*). G. Gene ontology analysis of the 1616 overlapping alternative splicing transcripts from (F) showing enrichment of critical transcript families. **H, I.** Representation of overlapping transcripts presenting with splicing changes in E7107 vs. vehicle, P5091 vs. vehicle and *shSF3B1#1* vs. control (H), *shSF3B1#2* vs. control (I). Analysis shows 1348 or 1237 common genes in all comparisons (*P<0.001*). **J.** Gene ontology analysis of the 1348 overlapping alternative splicing transcripts from (H) showing enrichment of critical transcript families. **K, L.** Density plot showing distance (log2) between pairs of 3' splice sites in *shSF3B1#2* (H) or E7107 treatment 24h (I). Blue line: downstream cryptic splice site. Red line: upstream cryptic splice site.

Figure S4. Han et al., 2020

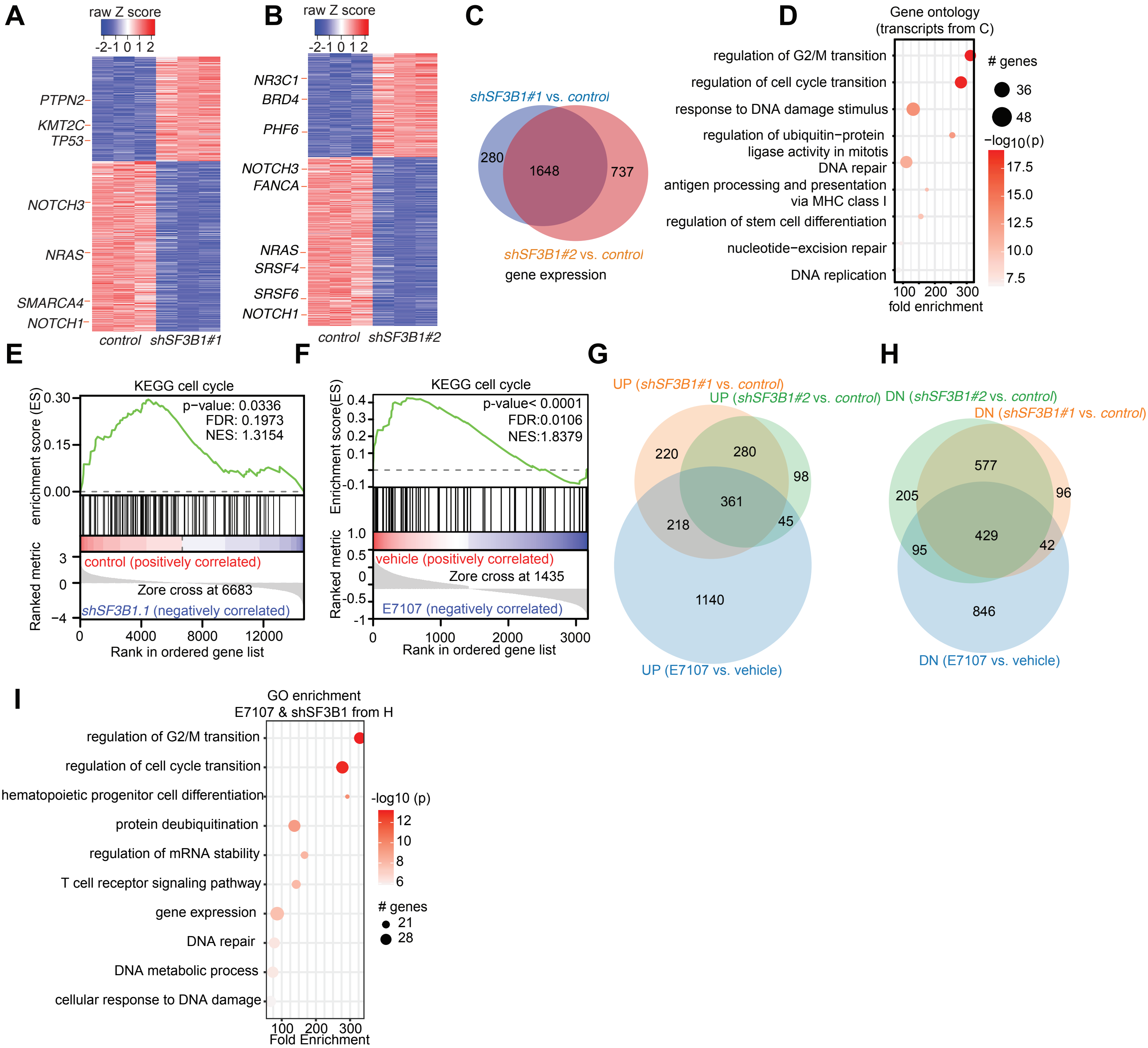

Supplementary Fig. 4. SF3B1 controls the levels of DDR transcripts. A. Heatmap of gene expression changes representing 784 significantly up-regulated genes and 1144 dn-regulated genes in *shSF3B1#1*-expressing CUTLL1 cells compared to *control* CUTLL1 cells (adj. $P<0.01$). **B.** Heatmap of gene expression changes representing 1079 significantly up-regulated genes and 1306 down-regulated genes in *shSF3B1#2*-expressing CUTLL1 cells compared to *control* CUTLL1 cells (adj. $P<0.01$). **C.** Overlap of differentially expressed transcripts in *control* vs. *shSF3B1#1* or *control* vs. *shSF3B1#2*. Analysis shows 1648 genes common in all comparisons ($P<0.001$). **D.** Gene ontology analysis of 1648 overlapping genes from (C) showing enrichment of critical transcript families, including cell cycle and DNA damage response pathways. **E, F.** GSEA showing cell cycle-associated transcript changes in *shSF3B1#1*-expressing vs. *control* CUTLL1 cells or (E) E7107 24h vs. vehicle-treated CUTLL1 cells (F). **G, H.** Overlap of up-regulated (UP) or down-regulated (DN) transcripts in *shSF3B1#1* vs. *control*, *shSF3B1#2* vs. *control* and E7107 vs. vehicle. Analysis shows the common genes in all comparisons ($P<0.001$). **I.** Gene ontology analysis of the overlapping transcripts from (H).

Figure S5. Han et al., 2020

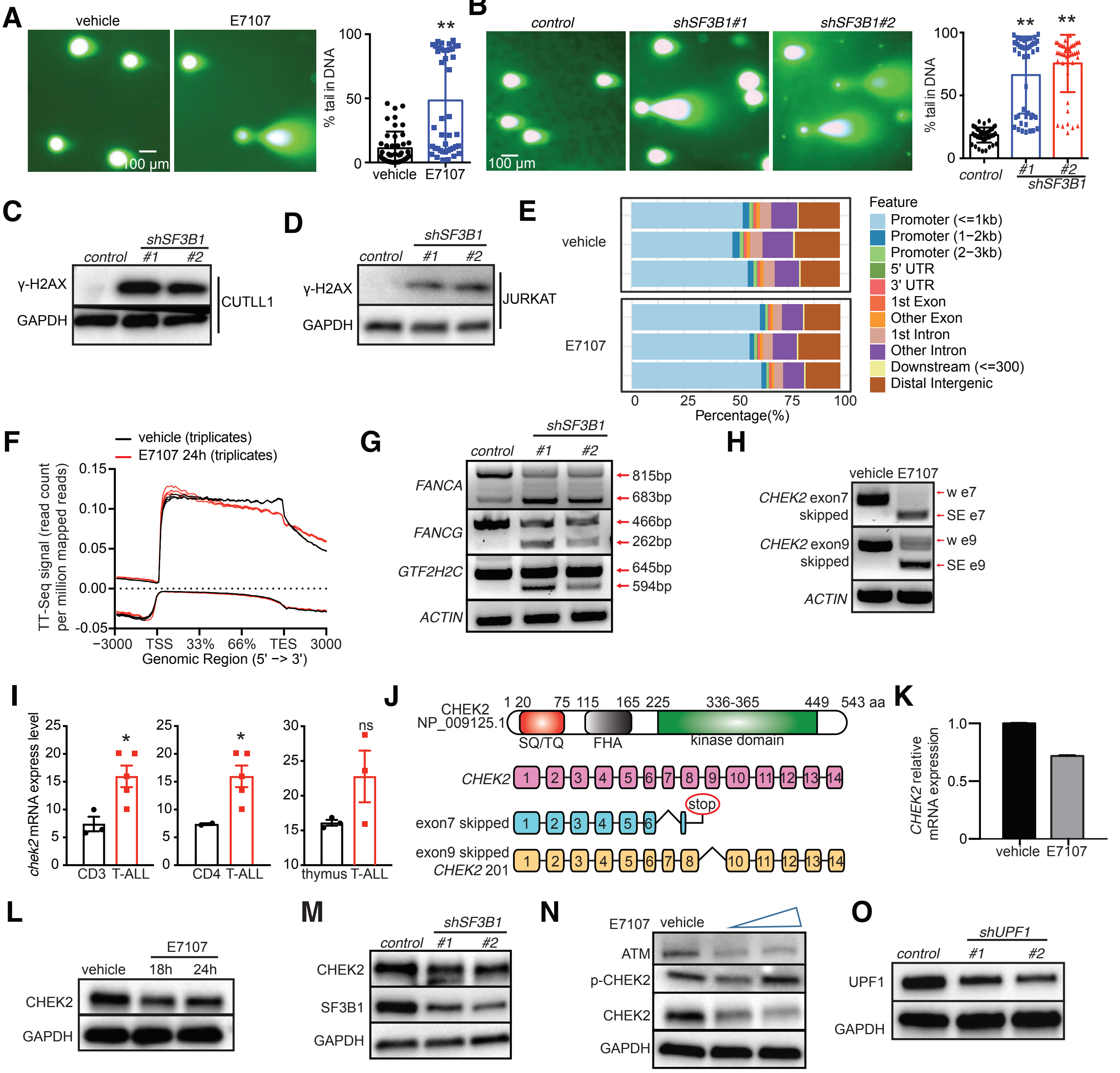

Supplementary Fig. 5. SF3B1 silencing or inhibition leads to CHEK2 exon skipping in T-ALL cell. A, B Representative photos (left) and quantification (right, n=45) of the percentage of tail DNA in total cellular DNA in neutral comet assay method in JURKAT cells treated with 3 nM E7107 for 48 h or CUTLL1 cells treated with control, *shSF3B1* are shown. **C.** Immunoblot analysis protein level as indicated in CUTLL1 cell line of control and *shSF3B1*. **D.** Immunoblot analysis protein level as indicated in JURKAT cells of control and *shSF3B1*. **E.** Percentage of R-Loop peak distribution in the genome upon vehicle or 3nM E7107 treatment for 24h in CUTLL1 cells (*n=3*). **F.** Metagene profiles of normalized TT-seq reads over the gene body of protein-coding transcripts (-3Kb to +3Kb) in cells treated with 3 nM E7107 for 24h. **G.** PCR detection of DDR transcripts-related exon skipping events of CUTLL1 cells control and *shSF3B1*. **H.** PCR detection of *CHEK2* exon 7/9 skipping in JURKAT cells treated with vehicle or 3 nM E7107 for 18 h (w e: with exon; SE: skipped exon). **I.** *CHEK2* mRNA expression analysis in T-ALL vs. CD3+, CD4+, or thymocytes (our RNA-Sequencing data). **J.** Schematic representation of human CHEK2 protein and domains. SQ/TQ, a regulatory domain containing multiple ATM/ ATR(ATX)-recognition sites; FHA, a fork-head-associated domain required for CHEK2 homo-dimerization and other protein-protein interactions. Amino acids 336 to 365 (in red) are encoded by exon 9 (top). Exon-exon junction schema for *CHEK2* with or without exon 7/9 skipping. **K.** PCR detection of *CHEK2* mRNA expression level in CUTLL1 cells treated with 3 nM E7107 for 24 h. **L.** Immunoblot analysis protein level for JURKAT cell line treated with vehicle or 3 nM E7107 for 18 h, 24 h. **M.** Immunoblot analysis of protein levels in control and *shSF3B1* JURKAT cells. **N.** Immunoblot analysis protein level as indicated in CUTLL1 cell line treated with vehicle, 1.5nM or 3 nM E7107 24 h. **O.** Immunoblot analysis in control or *shUPF1* CUTLL1 cells.

Figure S6. Han et al., 2020

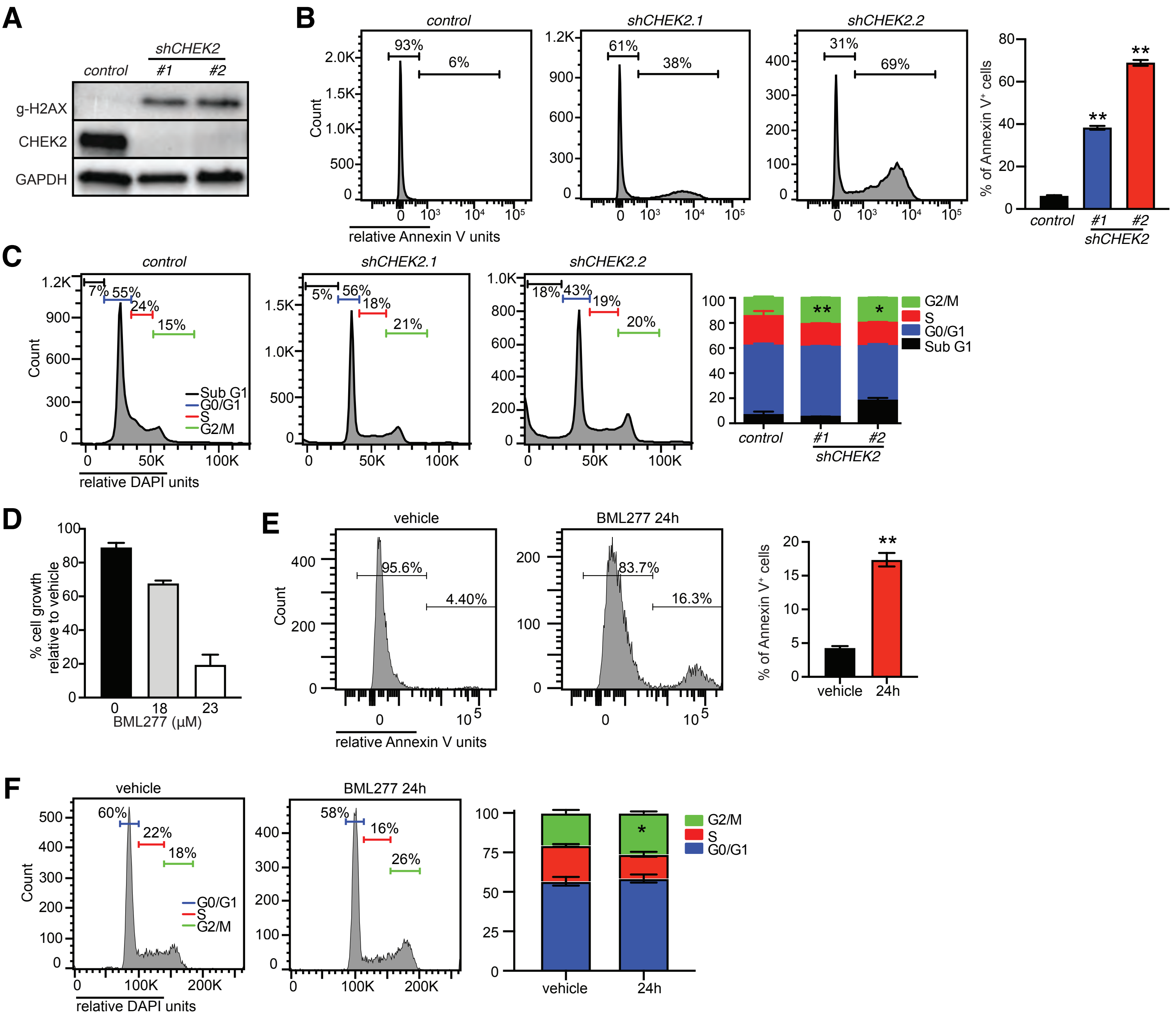

Supplementary Fig 6. CHEK2 is necessary for the growth of T-ALL. A. Immunoblot for the validation of *CHEK2* depletion in CUTLL1 cells. GAPDH is used as a loading control. **B.** Annexin V staining for *control*, *shCHEK2#1* and *shCHEK2#2*-expressing CUTLL1 cells 48 h post-puromycin selection. Representative plots of the annexin V percentage in the cell population assessed via flow-cytometry. Representative graphs depicting the percentages of annexin V-positive cells in the right (*n=3*). **C.** DAPI staining for *control*, *shCHEK2#1* and *shCHEK2#2*-expressing CUTLL1 cells 48 h post-puromycin selection (*n=3*). Quantification represents cell groups belonging to Sub-G1, G0/G1, S, G2/M phase populations (right). **D.** Represented cell growth relative to vehicle upon treatment with different concentrations of BML277. **E.** Annexin V staining for CUTLL1 cells treat with vehicle or 40 μM BML277 for 24 h. Representative plots of the annexin V percentage in the cell population assessed via flow-cytometry. Representative graphs depicting the percentages of annexin V-positive cells in the right panel (*n=3*). **F.** DAPI staining CUTLL1 cells treat with vehicle or 40 μM BML277 for 24 h (*n=3*). Quantification represents cell groups belonging to Sub-G1, G0/G1, S, G2/M phase populations (right).

Figure S7. Han et al., 2020

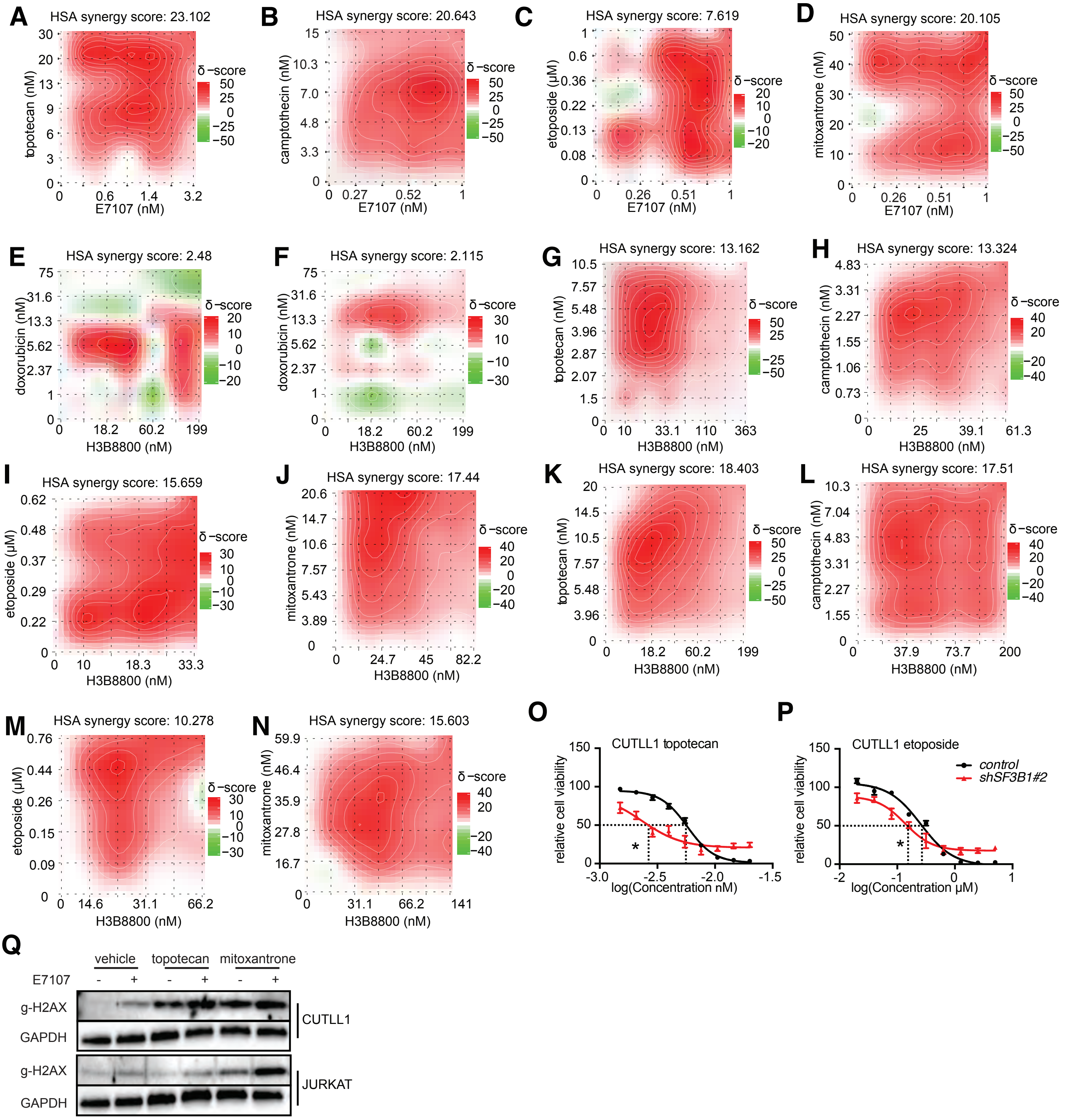

Supplementary Fig 7. SF3B1 inhibition can synergize with chemotherapy drugs towards blocking T-ALL growth. A-D. Synergy heatmaps for treatment with E7107 as well as topotecan, camptothecin, etoposide or mitoxantrone over a period of 3 days in JURKAT. Red color denotes drug synergy. **E-M.** Synergy heatmaps for combination treatments using H3B-8800 with doxorubicin, topotecan, camptothecin, etoposide or mitoxantrone over a period of 3 days in CUTLL1(E, G-J) or JURKAT (F, K-N)**. O-P.** IC50 analysis upon treatment of *control* and *shSF3B1#2* CUTLL1 cells with topotecan and etoposide. **Q.** Immunoblot analysis of γ-H2AX levels in CUTLL1 cells treated with vehicle or 3 nM E7107 with or without 50 nM topotecan or 50nM mitoxantrone (24 h). GAPDH is used as a loading control.

Figure S8. Han et al., 2020

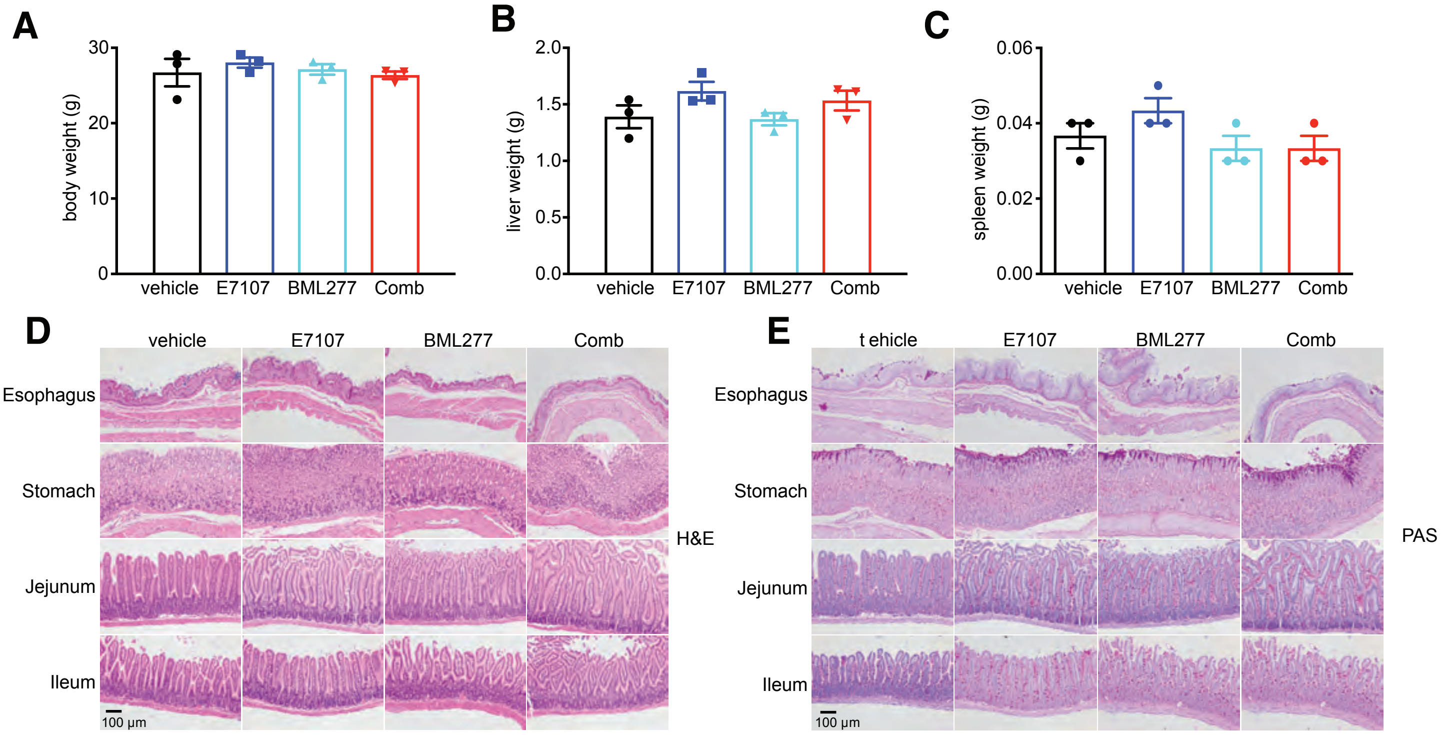

Supplementary Fig 8. SF3B1 or CHEK2 inhibition yields no toxicity in mice. A-C. Representative body, liver and spleen weight analysis upon treatment of E7107 only, BML277 only or E7107 and BML277 combination ("Comb"). **D, E.** Representative images of esophagus, stomach, jejunum and ileum. 200x magnification of H&E staining (D) and Periodic acid-Schiff (PAS) staining (E) (Please note that the photos used for the vehicle and E7107 conditions are the same as in Fig 1J and supplementary Fig 1I).
